# Restoration of m6A‐Modified circPSMB1 Suppresses Chordoma Progression and Enables Local Nanotherapeutic Intervention

**DOI:** 10.1002/advs.77417

**Published:** 2026-08-29

**Authors:** Yingchuang Tang, Liang Qiu, Xingbang Ruan, Yihan Shi, Ye Wang, Hanwen Li, Kai Zhang, Kangwu Chen

**Affiliations:** ^1^ Department of Orthopedic Surgery The First Affiliated Hospital of Soochow University Suzhou Jiangsu China

**Keywords:** chordoma, CircRNA, c‐MYC signaling, m6A modification, nanohydrogel therapy

## Abstract

Chordoma is a rare malignant bone tumor characterized by aggressive local invasion and high recurrence, yet its molecular regulatory mechanisms remain poorly understood. Circular RNAs (circRNAs) have emerged as important regulators of gene expression, but how circRNA epigenetic modifications contribute to oncogenic signaling is largely unknown. Here, we identify circPSMB1 as a significantly downregulated circRNA in chordoma tissues through high‐throughput circRNA sequencing. Functionally, circPSMB1 suppresses chordoma cell proliferation, migration, and invasion in vitro and inhibits tumor growth in vivo. Mechanistically, METTL3‐dependent m6A modification facilitates the association of circPSMB1 with the m6A reader HNRNPA2B1. CircPSMB1 competitively reduces the association of HNRNPA2B1 with c‐MYC mRNA, thereby decreasing c‐MYC protein expression without altering c‐MYC transcript abundance. Beyond mechanistic insight, we develop an injectable circPSMB1‐loaded HMnO_2_‐based nanohydrogel that enables sustained circRNA delivery and tumor microenvironment‐responsive therapy, effectively suppressing chordoma progression in vivo.

## Graphical abstract

An m6A‐modified circRNA, circPSMB1, is identified as a tumor suppressor in chordoma. METTL3‐dependent m6A promotes circPSMB1 interaction with HNRNPA2B1, limiting c‐MYC expression and malignant progression. An injectable HMnO_2_‐based nanohydrogel enables local circPSMB1 delivery and tumor microenvironment‐responsive therapy, offering a promising strategy for chordoma treatment.

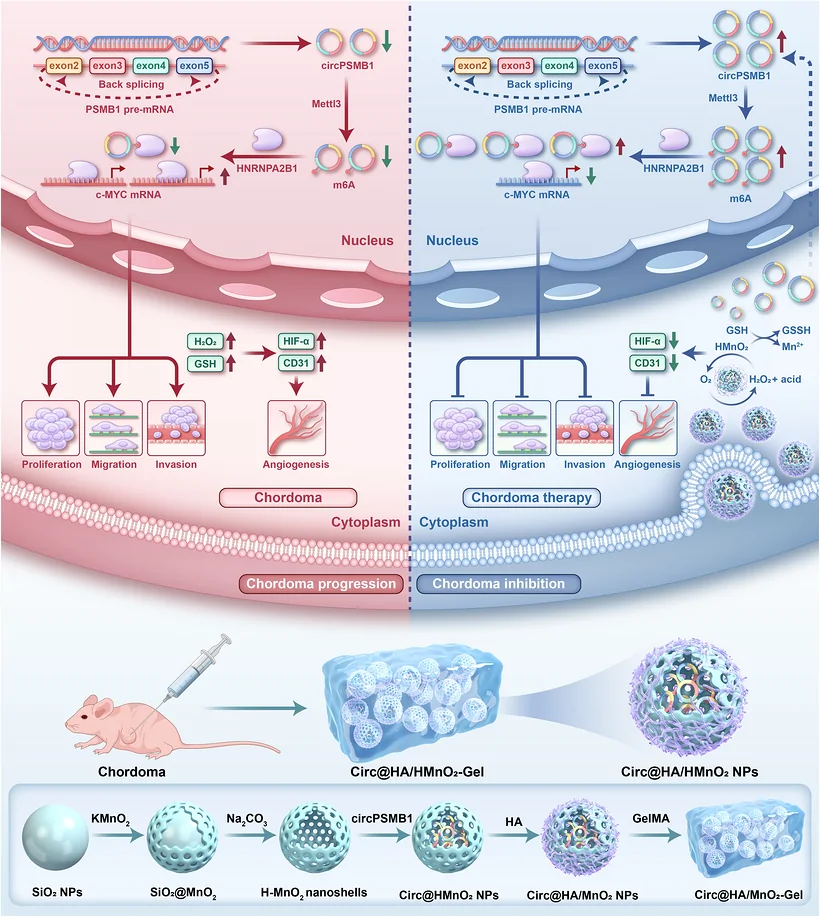

## 1 Introduction

Chordoma is a rare malignant bone tumor derived from embryonic notochord remnants and predominantly arises in the skull base, spine, and sacrococcygeal region [1, 2]. Despite its low incidence, chordoma poses significant clinical challenges due to its locally aggressive behavior, resistance to conventional chemotherapy and radiotherapy, and high postoperative recurrence rate [3]. Surgical resection remains the mainstay of treatment; however, complete resection is often difficult because of the tumor's anatomical location and infiltrative growth pattern [4]. Consequently, the long‐term prognosis of chordoma patients remains unsatisfactory, highlighting an urgent need to elucidate the molecular mechanisms underlying chordoma progression and to develop novel therapeutic strategies.

Circular RNAs (circRNAs) are a class of endogenous noncoding RNAs generated by back‐splicing of precursor mRNAs, resulting in covalently closed loop structures [5, 6]. Owing to their circular configuration, circRNAs are resistant to exonuclease‐mediated degradation and exhibit higher stability than linear RNAs [7]. Accumulating evidence indicates that circRNAs play critical roles in cancer initiation and progression by acting as microRNA sponges, interacting with RNA‐binding proteins (RBPs), regulating transcription, or modulating protein translation [8, 9, 10]. Dysregulated circRNAs have been implicated in multiple malignancies. For instance, Zhang et al. reported that circular RNA Circ_0007379 inhibits colorectal cancer progression by regulating miR‐320a biogenesis in a KSRP‐dependent manner [11]. Jiang et al. demonstrated the mechanistic role of circFNDC3B in regulating cancer cell metastatic properties and angiogenesis, suggesting that circFNDC3B may serve as a potential target for reducing OSCC metastasis [12]. Our previous study also preliminarily explored the role of circular TEAD1 in promoting chordoma tumorigenesis and progression by stabilizing Yap1 mRNA [13]. Nevertheless, the expression landscape and functional significance of circRNAs in chordoma remain largely unexplored.

N6‐methyladenosine (m6A) is the most prevalent internal modification in eukaryotic RNAs and dynamically regulates RNA metabolism, including splicing, stability, localization, and translation [14, 15]. The deposition of m6A is catalyzed by methyltransferase “writers” (such as METTL3), removed by “erasers” and interpreted by m6A “readers” including heterogeneous nuclear ribonucleoproteins [16]. Recent studies have revealed that m6A modification also occurs in circRNAs and profoundly influences their biological functions, particularly through mediating interactions with RBPs [17]. For example, Jin et al. untangled the interaction between m6A‐modified circTET2 and HNRNPC, which regulates fatty acid oxidation and promotes the proliferation of chronic lymphocytic leukemia [18]. Xie et al. discovered that N6‐methyladenosine modification of circNSUN2 facilitates its cytoplasmic export and stabilizes HMGA2, thereby promoting colorectal cancer liver metastasis [19]. However, the contribution of m6A‐modified circRNAs to chordoma progression remains unknown. To prioritize biologically relevant candidates identified by circRNA sequencing, KEGG enrichment analysis was performed using the parental genes of differentially expressed circRNAs. MAPK‐associated circRNAs were selected for initial validation as a candidate‐screening strategy [20, 21, 22], rather than as the mechanistic focus of this study.

**SCHEME 1:**
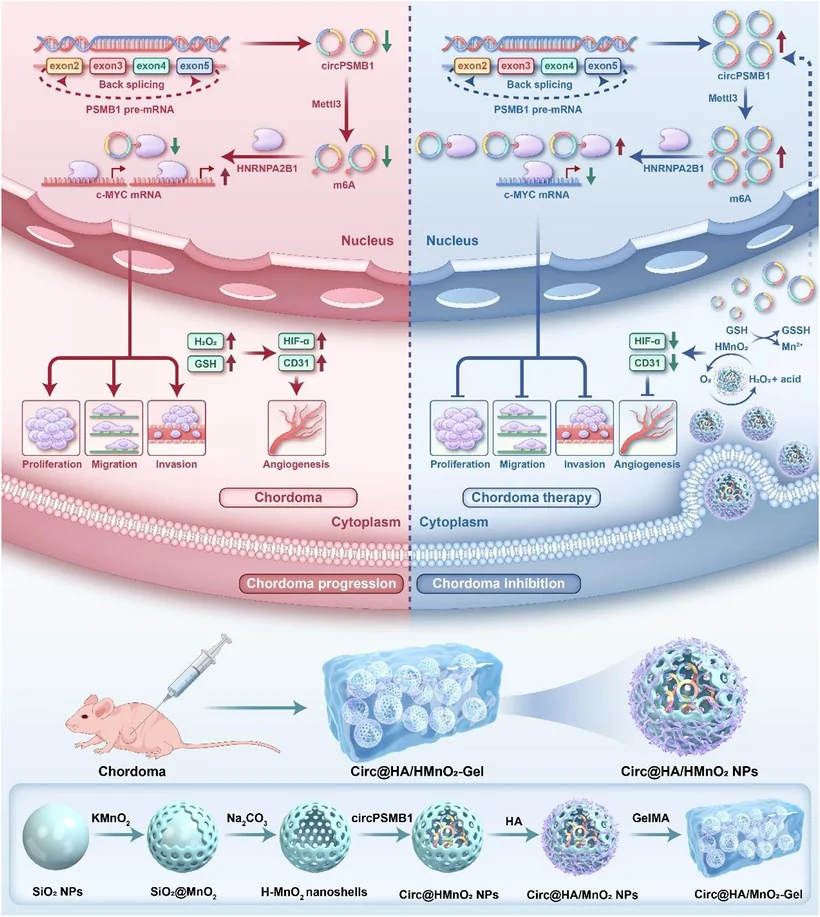
m6A‐modified circPSMB1 acts as a tumor suppressor in chordoma by competitively binding HNRNPA2B1 to regulate c‐MYC protein expression. An injectable circPSMB1‐loaded HMnO_2_‐based nanohydrogel enables local delivery and tumor microenvironment‐responsive release, effectively suppressing chordoma progression.

In this study, we performed high‐throughput circRNA sequencing to systematically characterize circRNA dysregulation in chordoma tissues and identified circPSMB1 as a significantly downregulated circRNA associated with patient prognosis. We further demonstrate that circPSMB1 suppresses chordoma cell proliferation, migration, and invasion by competitively binding to the m6A reader HNRNPA2B1 in a METTL3‐dependent manner, thereby reducing c‐MYC protein expression without affecting c‐MYC mRNA levels. Furthermore, to translate these mechanistic findings into a therapeutic strategy, we developed an injectable circPSMB1‐loaded nanohydrogel system (circ@HA/HMnO_2_‐Gel) that combines circRNA delivery with tumor microenvironment‐responsive catalytic therapy. This system exhibits favorable biosafety and potent antitumor efficacy both in vitro and in vivo. Collectively, our study reveals a previously unrecognized circRNA‐m6A‐RBP‐c‐MYC regulatory axis in chordoma and provides a promising nanotherapeutic strategy for chordoma treatment (Scheme 1).

## 2 Results

### 2.1 circPSMB1 is Downregulated in Chordoma

**FIGURE 1:**
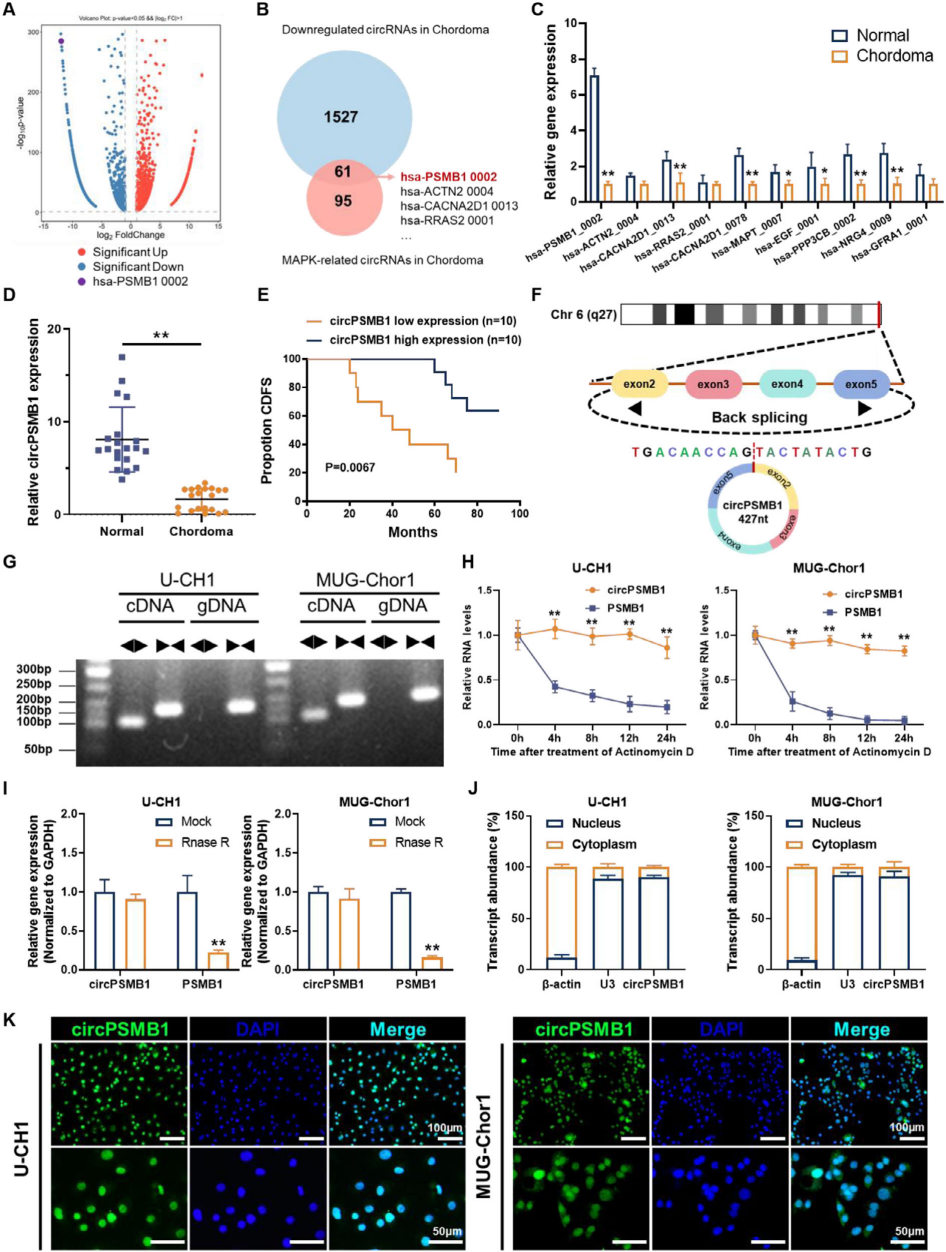
Identification of circPSMB1 downregulation and its molecular features in chordoma. (A) Volcano map illustrating differentially expressed circRNAs between normal and chordoma tissues. (B) Venn diagram depicting the intersection of circRNAs reduced in chordoma and those associated with the MAPK signaling pathway. (C) RT‐qPCR validation of the ten most markedly decreased MAPK‐related circRNAs in normal vs. chordoma specimens (*n* = 3 biologically independent samples). (D) RT‐qPCR quantification of circPSMB1 levels in normal and chordoma tissues (*n* = 20 biologically independent samples). (E) Continuous disease‐free survival (CDFS) stratified by circPSMB1 expression in chordoma patients. (F) Schematic showing that circPSMB1 (hsa_circ_0078784) originates from exons 2–5 of the PSMB1 gene and forms a 427‐nt circular transcript. (G) Agarose gel electrophoresis of PCR amplicons generated using divergent primers for circPSMB1 and convergent primers for linear PSMB1 in U‐CH1 and MUG‐Chor1 cells. (H) RT‐qPCR analysis of circPSMB1 and linear PSMB1 mRNA following actinomycin D exposure in U‐CH1 and MUG‐Chor1 cells (*n* = 3 biologically independent samples). (I) RT‐qPCR assessment of circPSMB1 and PSMB1 mRNA after RNase R digestion in U‐CH1 and MUG‐Chor1 cells (*n* = 3 biologically independent samples). (J) Nuclear‐cytoplasmic fractionation followed by RT‐qPCR detection of circPSMB1, with GAPDH and U3 serving as cytoplasmic and nuclear markers, respectively, in U‐CH1 and MUG‐Chor1 cells (*n* = 3 biologically independent samples). (K) RNA fluorescence in situ hybridization (FISH) analysis of circPSMB1 localization in U‐CH1 and MUG‐Chor1 cells, with nuclei counterstained by DAPI. *p < 0.05; **p < 0.01.

To investigate the role of circular RNAs (circRNAs) in the initiation and progression of chordoma, high‐throughput circRNA sequencing was performed on three paired chordoma tissues and adjacent non‐tumorous tissues. The results revealed that, compared with adjacent tissues, 1527 circRNAs were down‐regulated and 1461 circRNAs were up‐regulated in chordoma tissues (Figure 1A; Figure S1). Differentially expressed circRNAs were subsequently subjected to KEGG pathway enrichment analysis based on their parental genes. Among the significantly enriched pathways, MAPK‐associated circRNAs were selected as candidate molecules for further validation because of the established role of MAPK signaling in tumor progression. Based on this rationale, we further screened 45 downregulated circRNAs related to the MAPK signaling pathway (Figure 1B; pathway ID: hsa03050). Subsequently, real‐time quantitative polymerase chain reaction (RT‐qPCR) was employed to validate the relative expression levels of these candidate circRNAs. Among the top ten most significantly downregulated circRNAs, hsa‐PSMB1_0002 (hereafter referred to as circPSMB1) exhibited the most pronounced and consistent reduction in expression (fold change ≥ 10, *p* < 0.05) (Figure 1C). To further validate the RNA sequencing results, RT‐qPCR was performed to examine circPSMB1 expression in 20 chordoma tissues and 20 paired adjacent normal tissues. The results demonstrated that circPSMB1 expression was significantly reduced in chordoma tissues compared with adjacent normal tissues (Figure 1D). Moreover, low circPSMB1 expression in chordoma tissues was significantly associated with shorter continuous disease‐free survival (CDFS). Notably, follow‐up analysis revealed that patients with high circPSMB1 expression exhibited a significantly prolonged CDFS (Figure 1E). Collectively, these findings confirm that circPSMB1 is ubiquitously present but markedly downregulated in chordoma tissues and suggest a close association between circPSMB1 expression and patient prognosis.

### 2.2 Characteristics of circPSMB1 in Chordoma Cells

To determine the biogenesis of circPSMB1, we queried the circBank database (https://www.circbank.cn/) and found that circPSMB1 is a circular RNA originating from the PSMB1 locus on human chromosome 6. This circRNA is generated via back‐splicing between exons 2 and 5, resulting in a 427‐nt transcript (Figure 1F). To rule out potential confounding factors, such as trans‐splicing events, genomic rearrangements, or PCR‐derived artifacts that might falsely generate the circular junction, we designed convergent primers to amplify the linear PSMB1 mRNA and divergent primers to specifically detect circPSMB1. Agarose gel electrophoresis of the PCR products showed that circPSMB1 could be amplified by divergent primers in complementary DNA (cDNA) but not in genomic DNA (gDNA) (Figure 1G). Next, RNA stability assays were performed in chordoma cell lines (U‐CH1 and MUG‐Chor1) using actinomycin D treatment and RNase R digestion. Following actinomycin D treatment, circPSMB1 exhibited a significantly longer half‐life than linear PSMB1 mRNA (Figure 1H). Consistently, circPSMB1 displayed strong resistance to RNase R digestion compared with its linear counterpart. RNase R is a highly processive 3′‐5′ exoribonuclease that degrades linear RNAs, highlighting the enhanced stability conferred by the circular structure of circPSMB1 (Figure 1I). Together, these results confirm that circPSMB1 is more stable than linear PSMB1 mRNA. To determine the subcellular distribution of circPSMB1, we isolated nuclear and cytoplasmic RNA fractions from two chordoma cell lines and subsequently quantified circPSMB1 expression by RT‐qPCR. The results demonstrated that circPSMB1 was mainly located in the nucleus (Figure 1J), which was further validated by fluorescence in situ hybridization (FISH) assays (Figure 1K). In summary, these findings demonstrate that circPSMB1 is a highly abundant, stable circular RNA generated through back‐splicing of the PSMB1 gene, is significantly downregulated in chordoma, and may play an important role in chordoma tumorigenesis and progression.

### 2.3 circPSMB1 Inhibits the Migration and Proliferation of Chordoma Cells

**FIGURE 2:**
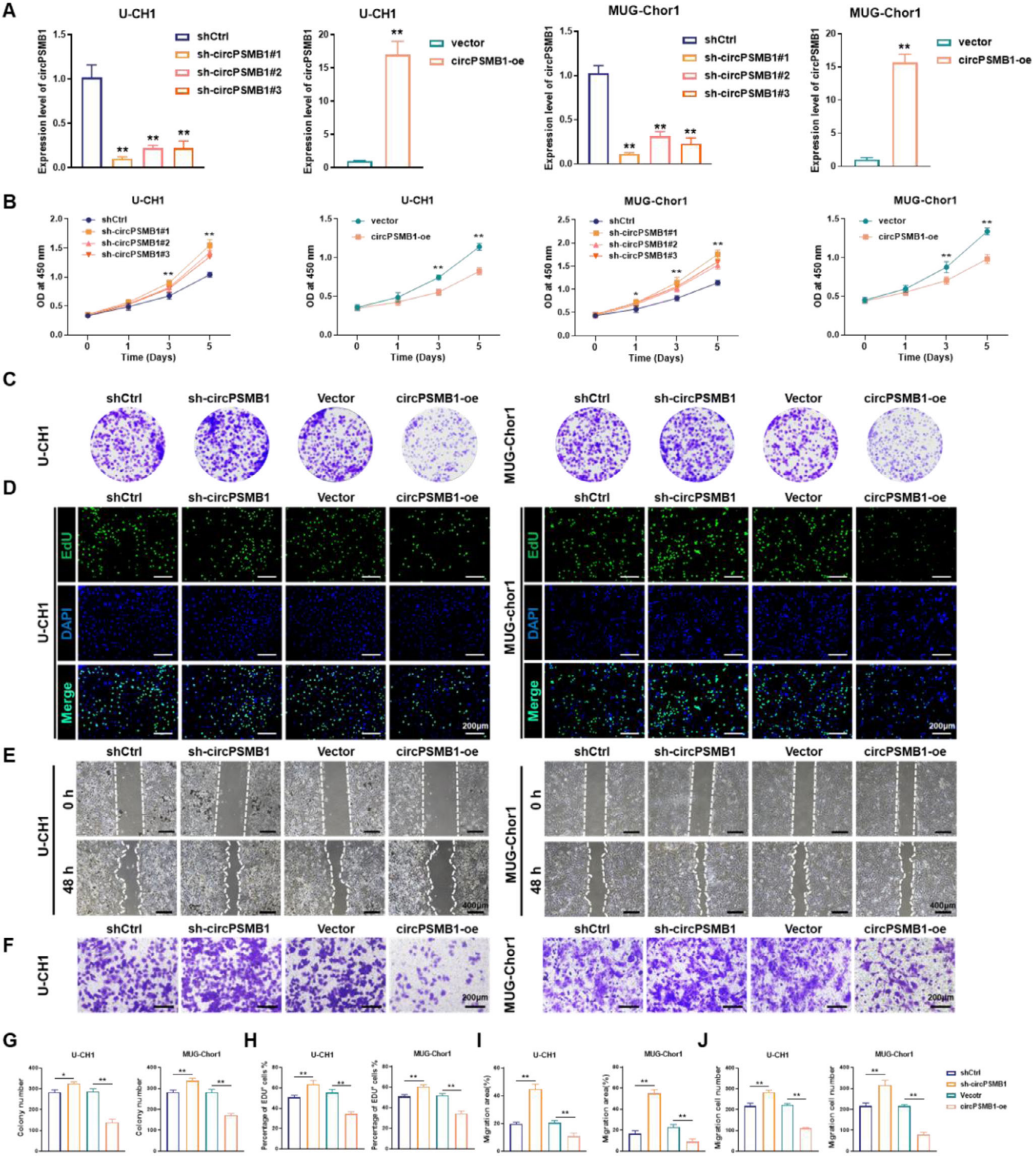
circPSMB1 suppresses chordoma cell proliferation and migration in vitro. (A) RT‐qPCR verification of circPSMB1 silencing and ectopic expression in two chordoma cell lines following transfection with sh‐circPSMB1 or oe‐circPSMB1, with shCtrl and empty vector as corresponding controls. (B) CCK‐8 assays evaluating cell viability after circPSMB1 knockdown or overexpression in U‐CH1 and MUG‐Chor1 cells (*n* = 3 biologically independent samples). (C) Colony formation assays assessing clonogenic capacity in response to circPSMB1 depletion or restoration. (D) EdU incorporation assays measuring DNA synthesis under altered circPSMB1 expression. (E) Wound‐healing assays examining the effects of circPSMB1 on migratory potential. (F) Transwell migration assays performed to determine cell motility following circPSMB1 knockdown or overexpression. (G) Statistical quantification of colony numbers from (C) (*n* = 3 independent experiments). (H) Quantification of EdU‐positive cells shown in (D) (*n* = 3 independent experiments). (I) Quantitative measurement of wound closure rates in (E) (*n* = 3 independent experiments). (J) Quantitative analysis of migrated cells from the Transwell assays in (F) (*n* = 3 independent experiments). *p < 0.05; **p < 0.01.

To better elucidate the biological importance of circPSMB1 in chordoma tumorigenesis and progression, two representative chordoma cell lines, U‐CH1 and MUG‐Chor1, were selected for subsequent functional studies. Three short hairpin RNAs specifically targeting the back‐splice junction of circPSMB1 (sh‐circPSMB1#1, #2, and #3; shCtrl as the control) and a circPSMB1 overexpression plasmid (oe‐circPSMB1; Vector as the control) were constructed and transfected into two chordoma cell lines. As expected, RT‐qPCR analysis demonstrated that sh‐circPSMB1 efficiently silenced circPSMB1 expression without affecting linear PSMB1 mRNA levels, whereas the overexpression plasmid increased circPSMB1 expression by more than tenfold, also without altering PSMB1 mRNA expression (Figure 2A). Although all three shRNAs achieved knockdown efficiencies exceeding 80% in both chordoma cell lines, sh‐circPSMB1#1 (hereafter referred to as sh‐circPSMB1) exhibited the strongest inhibitory effect and was therefore selected for subsequent experiments. Cell Counting Kit‐8 (CCK‐8) and colony formation assays revealed that circPSMB1 knockdown significantly promoted the proliferation of U‐CH1 and MUG‐Chor1 cells, whereas circPSMB1 overexpression markedly suppressed chordoma cell proliferation (Figure 2B,C). Consistently, EdU incorporation assays showed an increased proportion of EdU‐positive cells following circPSMB1 silencing, while circPSMB1 overexpression resulted in a reduced proportion of EdU‐positive cells in both cell lines (Figure 2D). Next, wound healing and Transwell assays were performed to assess the effects of circPSMB1 on chordoma cell migration. The results demonstrated that the migratory area of circPSMB1‐knockdown cells was significantly increased at 48 h compared with control cells, whereas circPSMB1 overexpression significantly reduced cell migration (Figure 2E). Similarly, Transwell assays showed that circPSMB1 knockdown increased the number of migrated cells, while circPSMB1 overexpression markedly decreased cell migration (Figure 2F). Quantitative analyses of colony formation, EdU incorporation, wound healing, and Transwell migration assays consistently illustrated the functional changes in chordoma cells following circPSMB1 knockdown or overexpression (Figure 2G–J). Furthermore, the expression level of PSMB1 mRNA was also examined and was not affected by circPSMB1‐specific shRNAs or the circPSMB1 overexpression vector (Figure S2). These findings indicate that the shRNAs used in this study do not degrade linear PSMB1 transcripts and that circPSMB1 does not enhance PSMB1 mRNA expression through previously reported positive feedback mechanisms. These findings indicate that circPSMB1 itself, rather than changes in linear PSMB1 mRNA expression, regulates chordoma cell proliferation and migration.

### 2.4 circPSMB1 Relies on METTL3‐Mediated m6A Modification to Interact with HNRNPA2B1 Suppressing Chordoma Progression

**FIGURE 3:**
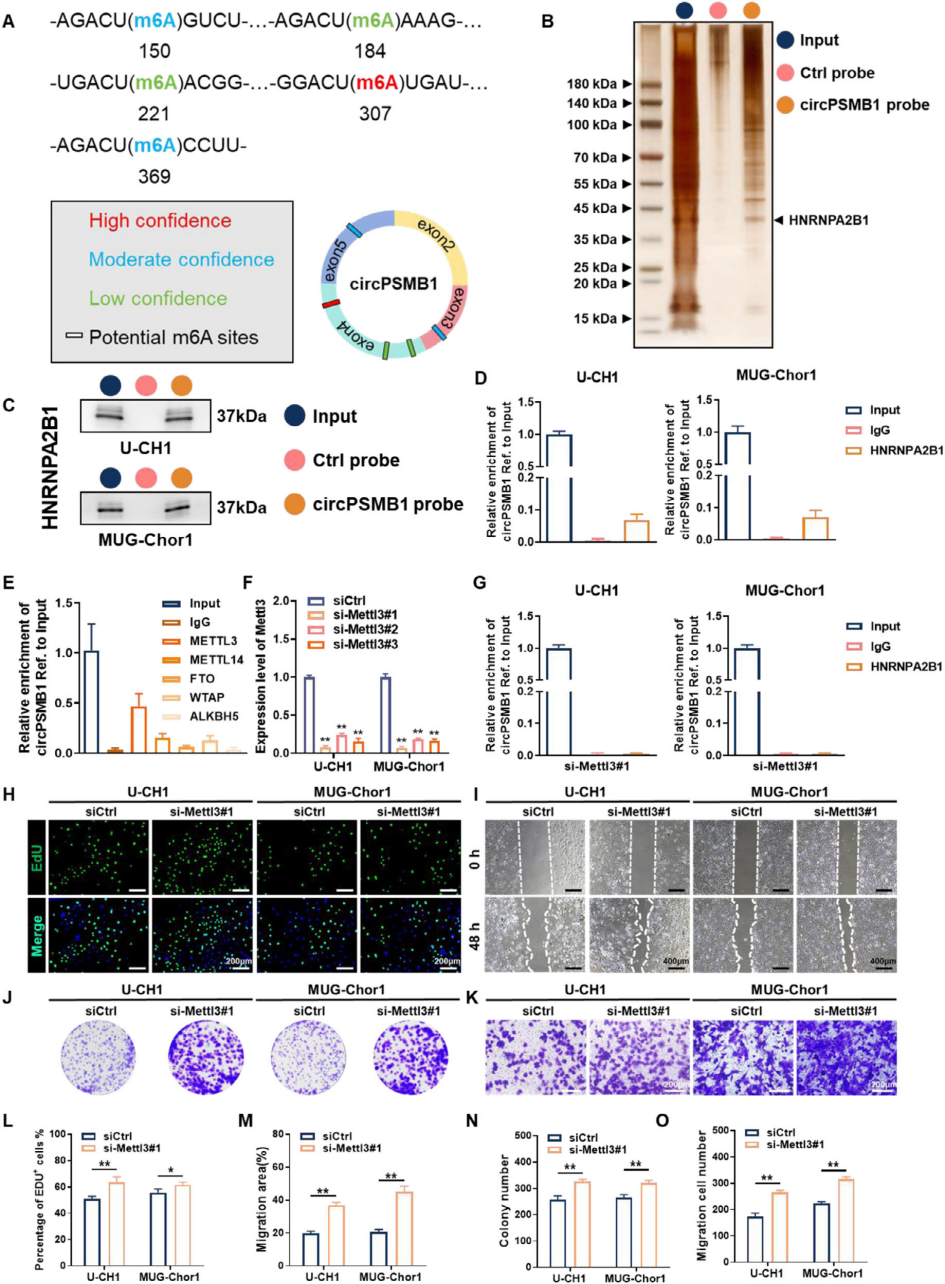
METTL3‐mediated m6A modification facilitates the association of circPSMB1 with HNRNPA2B1. (A) A schematic representation of the potential m6A‐modified sites in circPSMB1. (B) Characterization of circPSMB1‐associated proteins captured by a junction‐specific circPSMB1 probe using lysates from chordoma cells. (C) circPSMB1 pulldown assays in two chordoma cell lines. (D) RIP analysis using anti‐HNRNPA2B1 or control IgG to determine circPSMB1 enrichment in two chordoma cell lines. (E) RIP assays performed with antibodies against METTL3, METTL14, FTO, WTAP, and ALKBH5, with IgG as a negative control, to assess the binding of circPSMB1 to major m6A regulators. (F) RT‐qPCR validation of METTL3 silencing efficiency in two chordoma cell lines transfected with si‐METTL3 compared to siCtrl. (G) RIP assays using anti‐HNRNPA2B1 and IgG to evaluate circPSMB1 enrichment in two chordoma cell lines following METTL3 knockdown. (H) EdU assays assessing cell proliferation after METTL3 knockdown in two chordoma cell lines. (I) Wound‐healing assays evaluating migratory capacity in response to METTL3 knockdown. (J) Colony formation assays determining clonogenic growth following METTL3 knockdown. (K) Transwell migration assays performed to examine the effects of METTL3 silencing on cell motility. (L) Quantification of EdU‐positive cells shown in (H). (M) Statistical analysis of wound closure rates from (I). (N) Quantification of colony numbers derived from (J). (O) Quantitative evaluation of migrated cell numbers in (K). N = 3 biologically independent samples, *p < 0.05; **p < 0.01.

Recent studies have demonstrated that N6‐methyladenosine (m6A) modification is highly prevalent in both mRNAs and circular RNAs (circRNAs) and functionally regulates the eukaryotic transcriptome by influencing RNA splicing, nuclear export, localization, translation, and stability. To investigate whether circPSMB1 undergoes m6A modification, we first predicted potential m6A sites on circPSMB1 using the online bioinformatics tool SRAMP (Sequence‐based RNA Adenosine Methylation Site Predictor). A high‐confidence GGACU m6A consensus motif was identified within exon 4 of circPSMB1 (Figure 3A; Figure S3). Subsequently, electrophoresis, silver staining, and liquid chromatography‐tandem mass spectrometry (LC‐MS/MS) analyses were performed to identify proteins interacting with circPSMB1. Silver staining and screening results revealed that the m6A reader protein HNRNPA2B1 was a potential circPSMB1‐binding protein (Figure 3B). The interaction between circPSMB1 and HNRNPA2B1 was further validated by RNA pull‐down and RNA immunoprecipitation (RIP) assays. RNA pull‐down assays demonstrated that circPSMB1 efficiently enriched HNRNPA2B1 protein (Figure 3C). In addition, compared with IgG controls, RIP assays using an anti‐HNRNPA2B1 antibody showed significant enrichment of circPSMB1 in the immunoprecipitates, further supporting the association between circPSMB1 and HNRNPA2B1 (Figure 3D).

Because m6A methylation is controlled in a dynamic and reversible manner by methyltransferase “writers” and demethylase “erasers,” we conducted RIP assays in U‐CH1 cells to evaluate whether circPSMB1 interacts with key m6A regulators, including METTL3, METTL14, WTAP, FTO, and ALKBH5. The results showed that circPSMB1 was significantly enriched in the METTL3 immunoprecipitate, whereas no significant enrichment was detected in the RIP assays using antibodies against METTL14, WTAP, FTO, or ALKBH5 under our experimental conditions (Figure 3E). Although METTL14 is generally regarded as an essential component of the m6A methyltransferase complex, its association with circPSMB1 was not detectable by RIP in our experimental setting. Small interfering RNAs targeting METTL3 were subsequently constructed, and si‐METTL3#1 exhibited efficient knockdown in both U‐CH1 and MUG‐Chor1 cells (Figure 3F). Then we performed gene‐specific m6A RNA immunoprecipitation followed by RT‐qPCR (MeRIP‐qPCR) in both U‐CH1 and MUG‐Chor1 cells. The results demonstrated that circPSMB1 is significantly enriched in the m6A‐immunoprecipitated fraction, confirming that circPSMB1 undergoes m6A modification in chordoma cells. More importantly, METTL3 knockdown markedly reduced the m6A enrichment of circPSMB1 (Figure S4). To investigate whether m6A modification is required for HNRNPA2B1 recognition of circPSMB1, METTL3 was silenced or global RNA methylation was reduced using 3‐deazaadenosine (DAA). Both treatments markedly weakened the interaction between circPSMB1 and HNRNPA2B1, indicating that m6A modification is required for efficient HNRNPA2B1 recognition of circPSMB1 (Figure S5). To determine whether METTL3‐mediated m6A modification is functionally required for the interaction between circPSMB1 and HNRNPA2B1, RIP assays were repeated following METTL3 knockdown. The results demonstrated that METTL3 depletion markedly reduced the association between circPSMB1 and HNRNPA2B1 (Figure 3G). These findings indicate that circPSMB1 interacts with HNRNPA2B1 in a METTL3‐dependent, m6A‐mediated manner. To further investigate the functional role of METTL3 in chordoma, U‐CH1 and MUG‐Chor1 cells were transfected with si‐METTL3, and changes in proliferation, migration, and invasion were assessed using EdU incorporation assays (Figure 3H), wound healing assays (Figure 3I), colony formation assays (Figure 3J), and Transwell migration assays (Figure 3K). The results showed that METTL3 knockdown significantly enhanced chordoma cell proliferation, migration, and invasion (Figure 3L–O). Collectively, these findings establish that METTL3‐dependent m6A modification facilitates the association of circPSMB1 and HNRNPA2B1, providing the molecular basis for the tumor‐suppressive function of circPSMB1 in chordoma.

### 2.5 m6A‐Modified circPSMB1 Competitively Limits the Association of HNRNPA2B1 with c‐MYC mRNA

**FIGURE 4:**
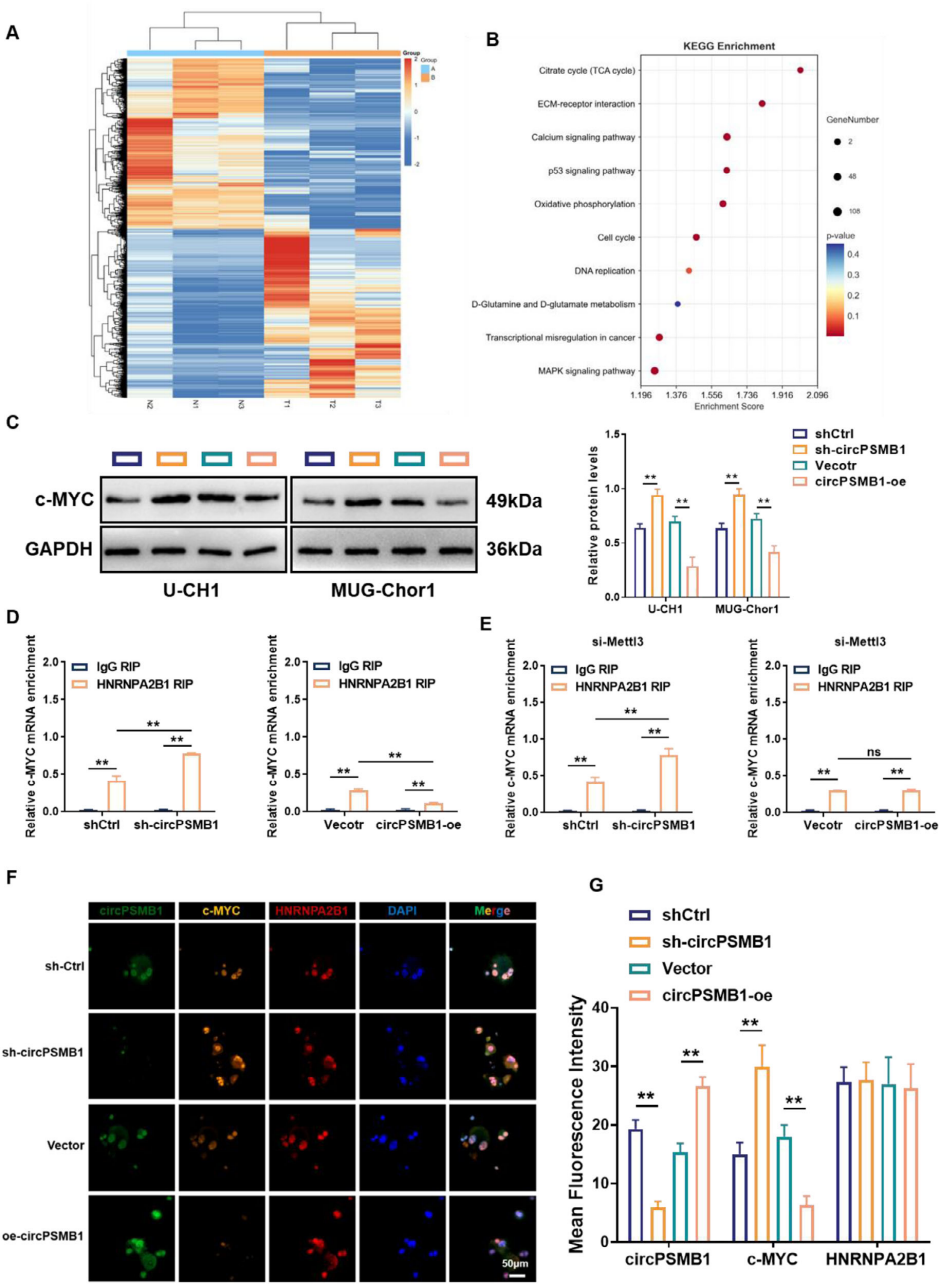
m6A‐modified circPSMB1 reduces c‐MYC protein expression by competitively limiting the association between HNRNPA2B1 and c‐MYC mRNA. (A) Heatmap showing the mRNA expression profiles and differentially expressed transcripts between normal and chordoma tissues. (B) KEGG enrichment analysis. (C) Western blot analysis of the c‐MYC proteins in U‐CH1 and MUG‐Chor1 cells transfected with sh‐circPSMB1 and circPSMB1‐oe. (D) RIP assays were conducted in U‐CH1 cells to assess the association of HNRNPA2B1 with c‐MYC mRNA following circPSMB1 silencing or ectopic expression. (E) The interaction between HNRNPA2B1 and c‐MYC mRNA was further examined by RIP in circPSMB1 knockdown or overexpression U‐CH1 cells under METTL3 depletion. (F) IF‐FISH assay of the colocalization of circPSMB1, c‐MYC and HNRNPA2B1. (G) Quantitative analysis of IF‐FISH assay in (F). N = 3 biologically independent samples, *p < 0.05; **p < 0.01.

To further investigate the regulatory interplay between circPSMB1 and HNRNPA2B1, RNA sequencing analysis was performed. Compared with adjacent normal tissues, a total of 617 differentially expressed mRNAs were identified in chordoma tissues, including 312 upregulated and 305 downregulated mRNAs. Hierarchical clustering analysis revealed that the two groups were clearly separated into distinct clusters, with mRNAs in each group exhibiting consistent expression patterns and regulatory directions (Figure 4A). Kyoto Encyclopedia of Genes and Genomes (KEGG) enrichment analysis of the target genes of downregulated mRNAs indicated that these genes were mainly involved in the regulation of cell proliferation, cell cycle progression, and other tumor‐related biological processes (Figure 4B). Recent studies have shown that aberrant activation or overexpression of the c‐MYC gene occurs in more than 70% of human cancers. As a master transcription factor, c‐MYC regulates critical cellular processes, including cell cycle progression, metabolism, and apoptosis. To determine whether circPSMB1 influences chordoma tumorigenesis by regulating c‐MYC expression, western blot analysis was performed to assess c‐MYC protein levels in U‐CH1 and MUG‐Chor1 cells following circPSMB1 knockdown or overexpression. The results demonstrated that circPSMB1 silencing markedly increased c‐MYC protein expression, whereas circPSMB1 overexpression significantly reduced c‐MYC protein levels (Figure 4C). In contrast, RT‐qPCR analysis confirmed that c‐MYC mRNA expression remained unchanged (Figure S6). These findings suggest that circPSMB1 regulates c‐MYC protein expression through an HNRNPA2B1‐dependent post‐transcriptional mechanism rather than by altering c‐MYC transcript abundance. Subsequently, RNA immunoprecipitation (RIP) assays revealed that following circPSMB1 knockdown, immunoprecipitation with an anti‐HNRNPA2B1 antibody resulted in a significant increase in the enrichment of c‐MYC mRNA in the precipitates. Conversely, circPSMB1 overexpression markedly reduced c‐MYC mRNA enrichment (Figure 4D). Importantly, when METTL3 was silenced while circPSMB1 was overexpressed, no statistically significant difference in c‐MYC mRNA enrichment was observed compared with the control group (Figure 4E). Furthermore, combined fluorescence in situ hybridization (FISH) and immunofluorescence (IF) staining demonstrated that circPSMB1 knockdown significantly increased c‐MYC expression, whereas circPSMB1 overexpression markedly reduced c‐MYC levels (Figure 4F). Notably, HNRNPA2B1 expression remained unchanged throughout these manipulations, indicating that HNRNPA2B1 functions as an RNA‐binding adaptor that mediates the interaction between circPSMB1 and c‐MYC mRNA rather than being transcriptionally regulated itself (Figure 4G; Figure S7A, B). Furthermore, rescue experiments were designed to validate the relationship between circPSMB1 and c‐MYC mRNA. Results from EdU staining and CCK‐8 assays indicated that the ability of circPSMB1 to inhibit chordoma cell proliferation was reversed upon co‐overexpression of circPSMB1 and c‐MYC. Furthermore, Transwell migration rescue assays demonstrated that restoration of c‐MYC expression significantly reversed the inhibitory effect of circPSMB1 on cell migration. These findings indicate that the tumor‐suppressive function of circPSMB1 is largely mediated through c‐MYC (Figure S8). Collectively, these findings indicate that m6A‐modified circPSMB1 competes with c‐MYC mRNA for HNRNPA2B1 association, thereby reducing c‐MYC protein expression through an HNRNPA2B1‐dependent post‐transcriptional mechanism. Functional rescue experiments further support c‐MYC as an important downstream effector of circPSMB1 in regulating chordoma cell proliferation and migration.

### 2.6 Characterization, Tumor Microenvironment‐responsive Release, and Biocompatibility of the circPSMB1@HA/HMnO_2_‐GelMA Hydrogel System

**FIGURE 5:**
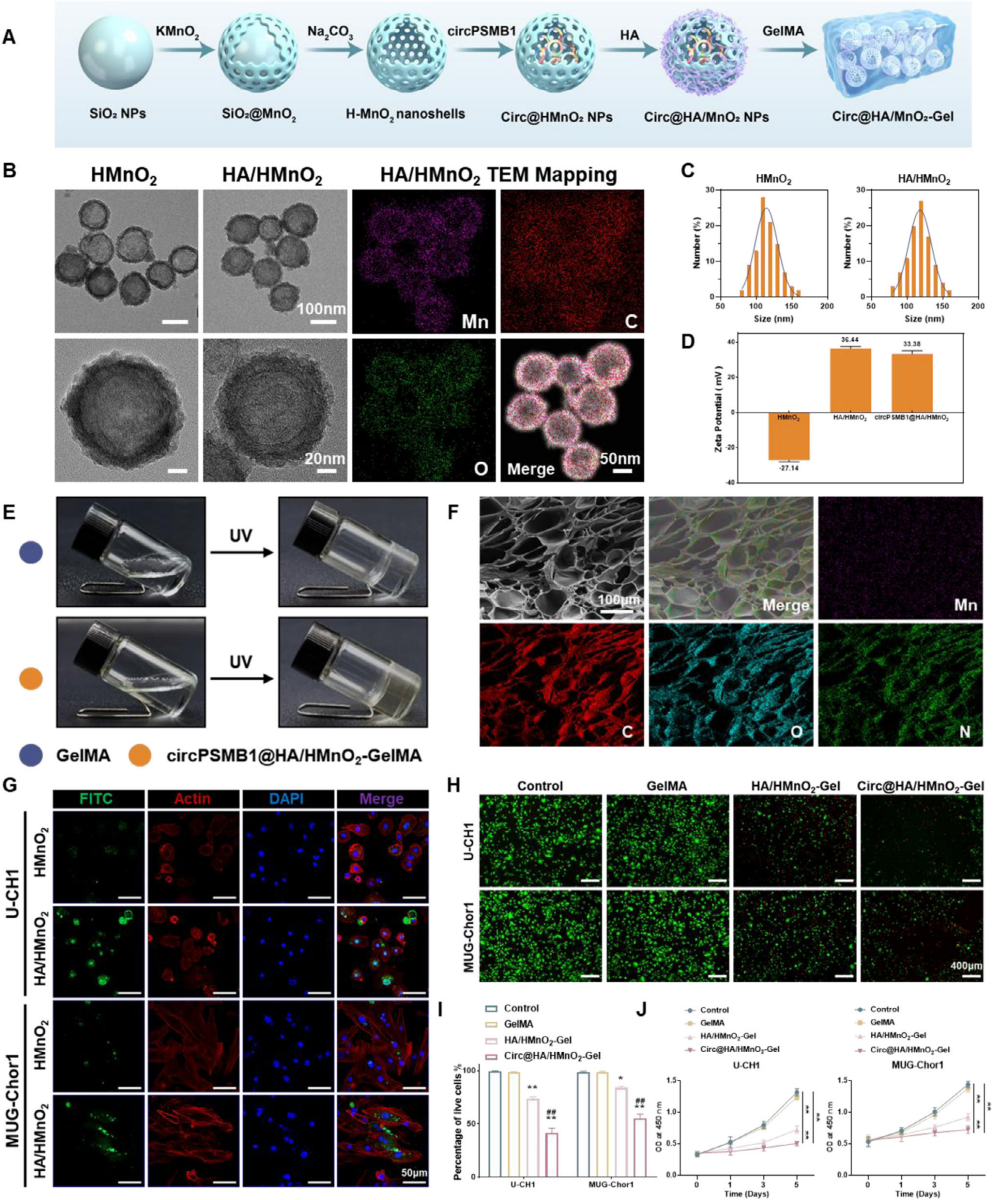
Characterization and biocompatibility of circPSMB1@HA/HMnO_2_‐GelMA hydrogel system. (A) Schematic illustration of the synthesis process of circPSMB1@HA/HMnO_2_‐GelMA hydrogel system. (B) TEM and elemental mapping images of HMnO_2_ and HA/HMnO_2_. (C) Particle size distribution analysis of HMnO_2_ and HA/HMnO_2_. (D) Zeta potential analysis of HMnO_2_, HA/HMnO_2_ and circPSMB1@HA/HMnO_2_. (E) Photographs of GelMA and circPSMB1@HA/HMnO_2_‐GelMA hydrogel system. (F) SEM and elemental mapping images of the circPSMB1@HA/HMnO_2_‐GelMA hydrogel. (G) Cellular uptake images of FITC‐labeled HMnO_2_ and HA/HMnO_2_ in two chordoma cell lines. (H) Live/Dead staining images of two chordoma cell lines co‐cultured with circPSMB1@HA/HMnO_2_‐GelMA hydrogel system. (I) Quantitative analysis of the percentage of live cells in (H). (J) CCK‐8 assay analysis in two chordoma cell lines co‐cultured with circPSMB1@HA/HMnO_2_‐GelMA hydrogel system. N = 3 biologically independent samples, *p < 0.05; **p < 0.01.

Given the pivotal role of circPSMB1 in chordoma, we next sought to develop a circPSMB1‐based nanodelivery system for local chordoma therapy. Manganese dioxide (MnO_2_) nanoparticles can react with glutathione (GSH) in the tumor microenvironment and be reduced to divalent manganese ions (Mn^2^^+^). Mn^2^^+^ catalyzes endogenous hydrogen peroxide (H_2_O_2_) within tumors to generate hydroxyl radicals (·OH), thereby inducing oxidative stress and promoting tumor cell apoptosis. In addition, hollow mesoporous manganese dioxide (HMnO_2_) nanostructures, characterized by a mesoporous shell and large internal cavity, have been demonstrated to be reliable carriers for enzymes and macromolecules, providing an ideal platform for high‐capacity circPSMB1 loading. Accordingly, HMnO_2_ nanoshells were employed as delivery vehicles for circPSMB1. HMnO_2_ nanoshells were synthesized following previously reported protocols. Briefly, solid silica (sSiO_2_) nanoparticles were first prepared using the classical Stöber method as hard templates. These particles were then reacted with potassium permanganate (KMnO_4_) to form a uniform MnO_2_ coating on the surface (sSiO_2_‐MnO_2_). Subsequently, the sSiO_2_ cores were selectively etched using sodium carbonate (Na_2_CO_3_) solution to obtain hollow HMnO_2_ nanoshells. CircPSMB1 was then encapsulated into the HMnO_2_ nanoshells via co‐incubation to generate circPSMB1@HMnO_2_, followed by surface modification with hyaluronic acid (HA) to obtain circPSMB1@HA/HMnO_2_. HA coating enhanced nanoparticle stability, reduced premature cargo leakage, and may potentially improve local retention. To improve injectability, circPSMB1@HA/HMnO_2_ was mixed with GelMA to form the final injectable hydrogel delivery system, circPSMB1@HA/HMnO_2_‐GelMA (abbreviated as circ@HA/HMnO_2_‐Gel), which was used for subsequent experiments (Figure 5A). Transmission electron microscopy (TEM) and elemental mapping analyses confirmed the successful synthesis of HMnO_2_ nanoshells and HA/HMnO_2_ nanoparticles (Figure 5B). The average particle sizes of HMnO_2_ and HA/HMnO_2_ were 115.7 and 118.0 nm, respectively (Figure 5C). The zeta potentials of HMnO_2_, HA/HMnO_2_, and circ@HA/HMnO_2_ were ‐27.14, 36.44, and 33.38 mV, respectively (Figure 5D).

To assess whether incorporation of circPSMB1@HA/HMnO_2_ nanoparticles affected the physicochemical properties of the GelMA hydrogel, a series of rheological and structural characterizations were performed. Frequency sweep rheological analysis demonstrated that both GelMA and circPSMB1@HA/HMnO_2_‐GelMA hydrogels exhibited storage moduli (G′) consistently higher than loss moduli (G″) across the tested frequency range, indicating stable gel‐like behavior. Importantly, incorporation of circPSMB1@HA/HMnO_2_ caused no obvious changes in the viscoelastic properties of the GelMA hydrogel (Figure S9). Swelling and degradation assays further demonstrated that the composite hydrogel retained physicochemical characteristics comparable to those of GelMA alone, exhibiting similar swelling kinetics and gradual degradation profiles (Figure S10). To evaluate the responsiveness of the hydrogel to the tumor microenvironment, cumulative release profiles of Mn^2^^+^ and circPSMB1 were determined under different pH and glutathione (GSH) conditions. Under physiological conditions (pH 7.4), only minimal release was observed. In contrast, acidic conditions (pH 5.5) markedly accelerated cargo release, which was further enhanced in the presence of 10 mM GSH. More than 90% of the loaded Mn^2^^+^ and approximately 85–90% of circPSMB1 were released within 24 h under pH 5.5 supplemented with 10 mM GSH, confirming the pH/GSH‐responsive release behavior of the circPSMB1@HA/HMnO_2_‐GelMA hydrogel (Figure S11).

Morphological analyses further revealed that incorporation of circPSMB1@HA/HMnO_2_ did not affect the sol‐gel transition behavior of GelMA (Figure 5E). Scanning electron microscopy (SEM) and elemental mapping analyses of circ@HA/HMnO_2_‐Gel demonstrated a loose and porous architecture with circ@HA/HMnO_2_ nanoparticles uniformly distributed throughout the GelMA matrix (Figure 5F). To minimize cytotoxicity toward normal cells, appropriate concentrations of HMnO_2_ were determined using CCK‐8 assays and live/dead staining. CCK‐8 results indicated that HMnO_2_ or HA/HMnO_2_ concentrations exceeding 100 µg/mL induced significant cytotoxicity in nucleus pulposus cells (Figure S12A). When nucleus pulposus cells were co‐cultured with 80 µg/mL HMnO_2_ or HA/HMnO_2_ for 1, 3, and 5 days, live/dead staining revealed no obvious cytotoxic effects (Figure S12B).

To evaluate nanoparticle uptake by chordoma cells, FITC‐labeled bovine serum albumin (BSA) was loaded into HMnO_2_ and HA/HMnO_2_ nanoparticles to monitor cellular internalization. The results showed that both U‐CH1 and MUG‐Chor1 cells exhibited significantly greater uptake of HA/HMnO_2_ nanoparticles compared with HMnO_2_ nanoparticles (Figure 5G; Figure S13), indicating that HA modification enhances nanoparticle retention and cellular uptake in chordoma cells. Next, the antitumor efficacy of circ@HA/HMnO_2_‐Gel was evaluated using live/dead staining and CCK‐8 assays. After co‐culturing two chordoma cell lines with GelMA, HA/HMnO_2_‐Gel, or circ@HA/HMnO_2_‐Gel for 5 days, live/dead staining revealed that GelMA exhibited effects comparable to the control group, whereas both HA/HMnO_2_‐Gel and circ@HA/HMnO_2_‐Gel displayed significant antitumor activity. Notably, circ@HA/HMnO_2_‐Gel demonstrated the most pronounced antitumor effect (Figure 5H). Quantitative analysis of live cells and CCK‐8 assays further substantiated these findings (Figure 5I,J). Taken together, these results demonstrate the successful construction of the circ@HA/HMnO_2_‐Gel hydrogel delivery system, which exhibits favorable biosafety and potent antitumor efficacy in vitro.

### 2.7 circPSMB1@HA/HMnO_2_‐GelMA Hydrogel System Inhibits the Migration and Proliferation of U‐CH1 and MUG‐Chor1 cells

**FIGURE 6:**
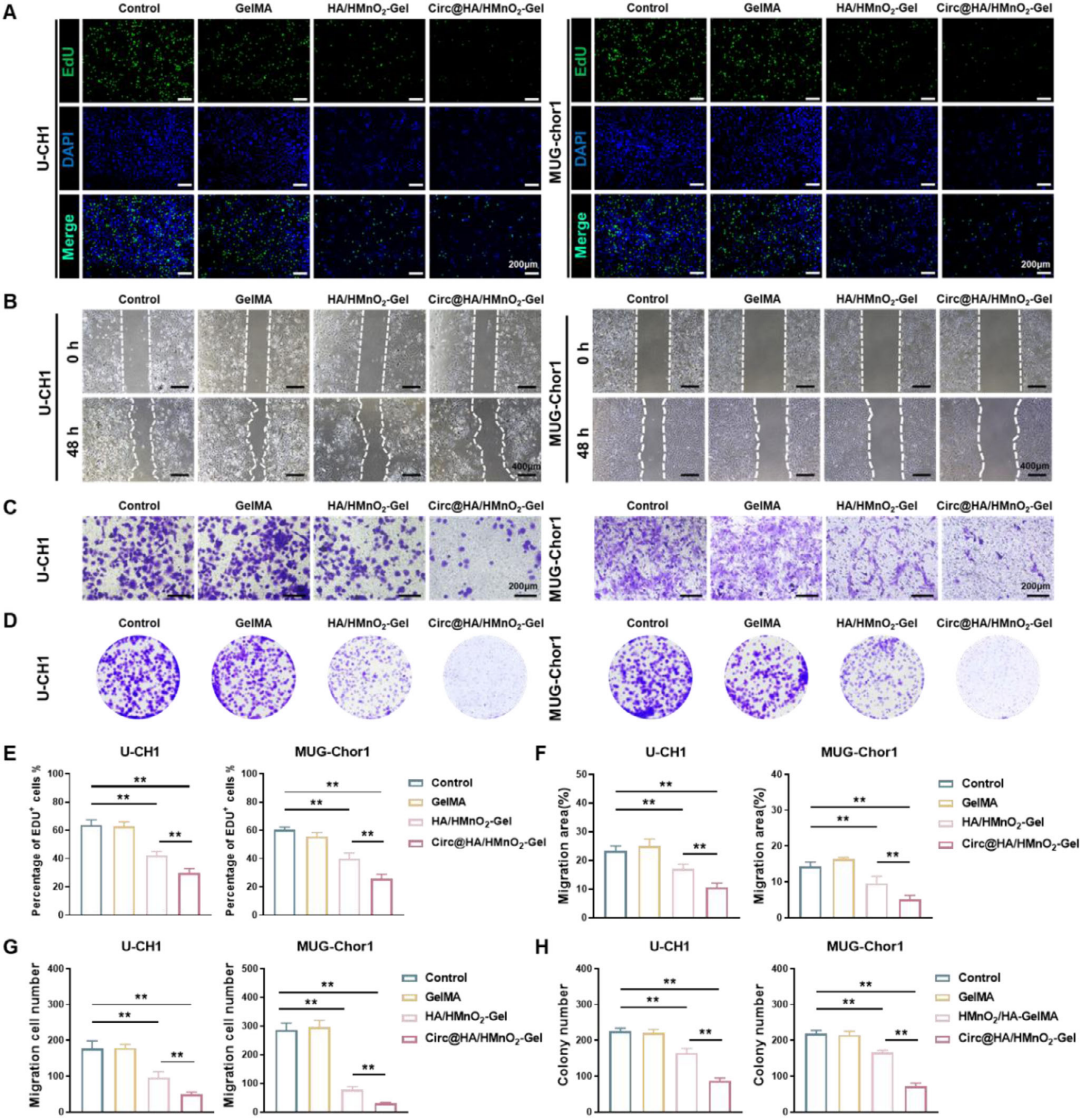
circPSMB1@HA/HMnO_2_‐GelMA hydrogel system inhibits the migration and proliferation of U‐CH1 and MUG‐Chor1 cells. (A) Wound‐healing assays performed in two chordoma cell lines to evaluate migratory capacity. (B) EdU incorporation assays in two chordoma cell lines to assess DNA synthesis and proliferation. (C) Transwell migration assays conducted using two chordoma cell lines. (D) Colony formation experiments carried out in two chordoma cell lines to determine clonogenic growth. (E) Statistical quantification of wound closure rates corresponding to (A). (F) Quantitative evaluation of EdU‐positive cells from (B). (G) Quantification of migrated cells in the Transwell assays shown in (C). (H) Summary statistics of colony numbers derived from (D). N = 3 independent experiments, *p < 0.05; **p < 0.01.

To confirm the impact of the circ@HA/HMnO_2_‐Gel hydrogel system on chordoma progression, two chordoma cell lines were co‐cultured with GelMA, HA/HMnO_2_‐Gel, or circ@HA/HMnO_2_‐Gel. EdU incorporation, wound healing, Transwell, and colony formation assays were used to assess changes in chordoma cell migration, proliferation, and invasion. EdU assays demonstrated that, in both chordoma cell lines, the proportion of EdU‐positive cells showed no statistically significant difference between the GelMA group and the control group. In contrast, the proportion of EdU‐positive cells was significantly reduced in the HA/HMnO_2_‐Gel and circ@HA/HMnO_2_‐Gel groups, with the lowest proportion observed in the circ@HA/HMnO_2_‐Gel group, which was significantly lower than that in the HA/HMnO_2_‐Gel group (Figure 6A). Wound healing assays revealed that the migratory areas of cells in the HA/HMnO_2_‐Gel and circ@HA/HMnO_2_‐Gel groups were significantly smaller than those in the control group at 48 h, with circ@HA/HMnO_2_‐Gel exhibiting a more pronounced inhibitory effect compared with HA/HMnO_2_‐Gel (Figure 6B). Consistently, Transwell assays showed that circ@HA/HMnO_2_‐Gel markedly reduced the number of migrated cells, with significant differences observed compared with all other groups (Figure 6C). Colony formation assays further confirmed that circ@HA/HMnO_2_‐Gel significantly suppressed the proliferative capacity of chordoma cells (Figure 6D). Quantitative analyses clearly illustrated the differences among the experimental groups (Figure 6E‐H). Collectively, these results demonstrate that the circ@HA/HMnO_2_‐Gel hydrogel system effectively inhibits chordoma cell migration, proliferation, and invasion.

### 2.8 Efficacy and safety of the circPSMB1@HA/HMnO_2_‐GelMA Hydrogel System in Inhibiting Chordoma in Vivo

**FIGURE 7:**
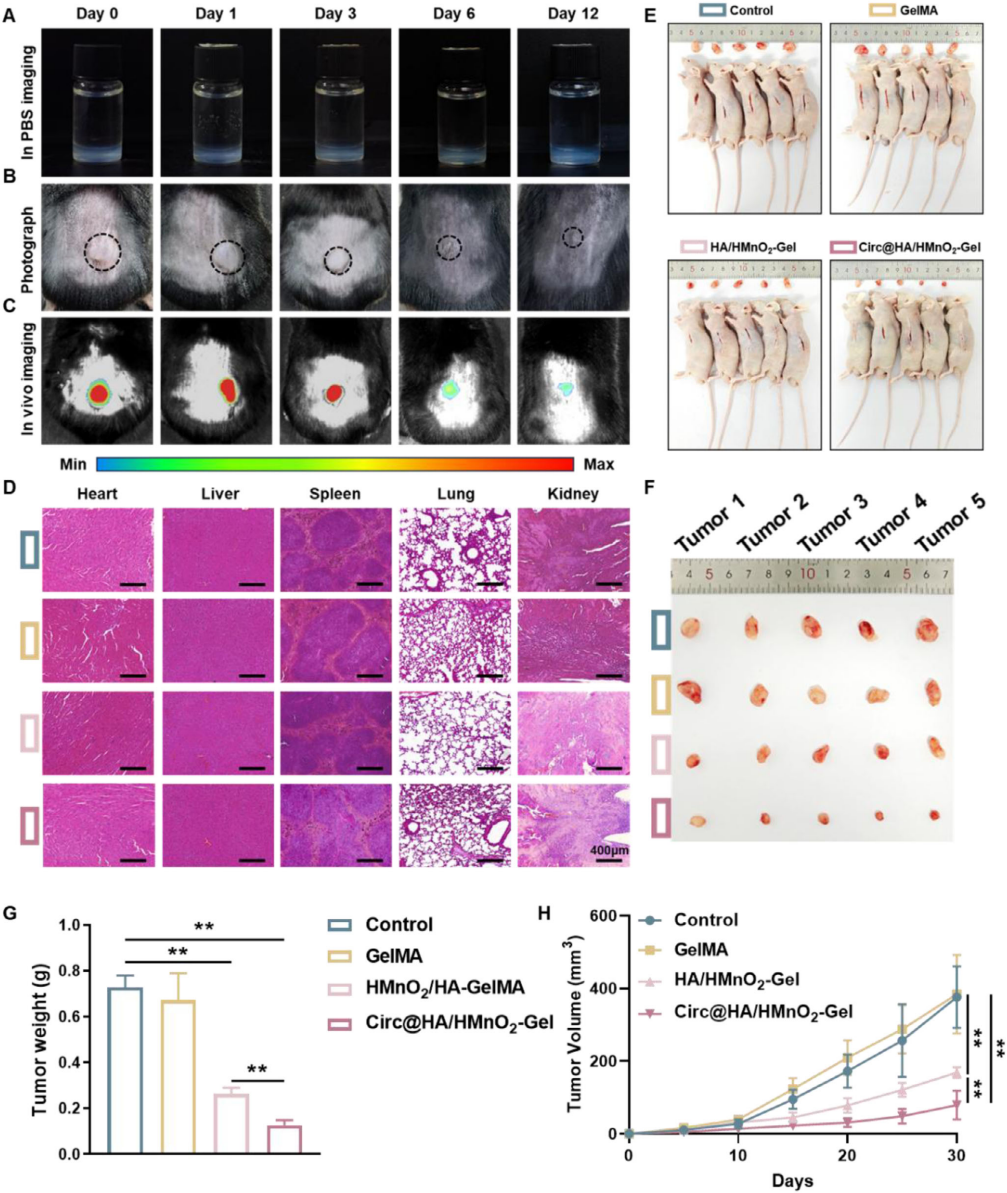
Efficacy and safety of the circPSMB1@HA/HMnO_2_‐GelMA hydrogel system in inhibiting chordoma in vivo. (A) Representative photographs showing the in vitro degradation behavior of circ@HA/HMnO_2_‐Gel in PBS at different time points. (B) In vivo photographs showing the macroscopic degradation behavior of the in situ formed circ@HA/HMnO_2_‐Gel at healthy C57/BL6 mice. (C) In vivo degradation profile of Cy3‐labeled circ@HA/HMnO_2_‐Gel recorded by fluorescence imaging within 12 days. (D) Representative images of H&E‐stained sections of major organs isolated from mice on day 12 after receiving different treatments. (E) Treatment of nude mice bearing subcutaneously implanted U‐CH1 cells with the circ@HA/HMnO_2_‐Gel hydrogel system. (F) Representative excised tumors from each experimental group are shown. (G) Tumor volume was monitored over time, and growth rates are presented for the indicated groups. (H) Final tumor weights in different groups. N = 5 independent mice, *p < 0.05; **p < 0.01.

To further evaluate the biosafety and therapeutic efficacy of the circ@HA/HMnO_2_‐Gel hydrogel system, its degradation behavior and toxicity were assessed in C57BL/6 mice, and its antitumor efficacy was validated in nude mouse models. In vitro degradation studies showed that circ@HA/HMnO_2_‐Gel maintained structural integrity for at least 12 days at 37°C, followed by gradual degradation until day 28 (Figure 7A). To investigate in vivo degradation, 150 µL of circ@HA/HMnO_2_‐Gel solution was subcutaneously injected into the dorsal region of healthy C57BL/6 mice. As shown in Figure 7B, the size of the in situ‐formed circ@HA/HMnO_2_‐Gel gradually decreased over time, and no visible hydrogel nodules were observed at the injection site by day 12 post‐injection. To further characterize in vivo degradation behavior, Cy3 was incorporated as a fluorescent tracer into the circ@HA/HMnO_2_‐Gel and injected subcutaneously into the abdominal region of mice. In vivo fluorescence imaging revealed that the Cy3‐labeled circ@HA/HMnO_2_‐Gel hydrogel remained detectable for at least 12 days (Figure 7C). Moreover, hematoxylin and eosin (H&E) staining of major organs collected from C57BL/6 mice 28 days after subcutaneous injection of circ@HA/HMnO_2_‐Gel showed no significant inflammatory responses (Figure 7D), indicating favorable biodegradability and biocompatibility of the hydrogel system. Next, a chordoma subcutaneous xenograft model was established in nude mice to evaluate the in vivo antitumor efficacy of circ@HA/HMnO_2_‐Gel. Two weeks after subcutaneous implantation of U‐CH1 cells, mice received intratumoral injections of saline, GelMA, HA/HMnO_2_‐Gel, or circ@HA/HMnO_2_‐Gel (Figure 7E). Tumor volumes were measured every 5 days starting from day 5, and all mice were sacrificed on day 30 for tumor excision and analysis. Notably, mice treated with circ@HA/HMnO_2_‐Gel exhibited significantly reduced tumor volumes and weights compared with the saline control, GelMA, and HA/HMnO_2_‐Gel groups (Figure 7F–H). Taken together, these findings demonstrate that circ@HA/HMnO_2_‐Gel possesses favorable biosafety and robust antitumor efficacy in vivo.

### 2.9 Histological Evaluation of the circPSMB1@HA/HMnO_2_‐GelMA Composite Hydrogel System Demonstrates Therapeutic Efficacy in Chordoma Models

**FIGURE 8:**
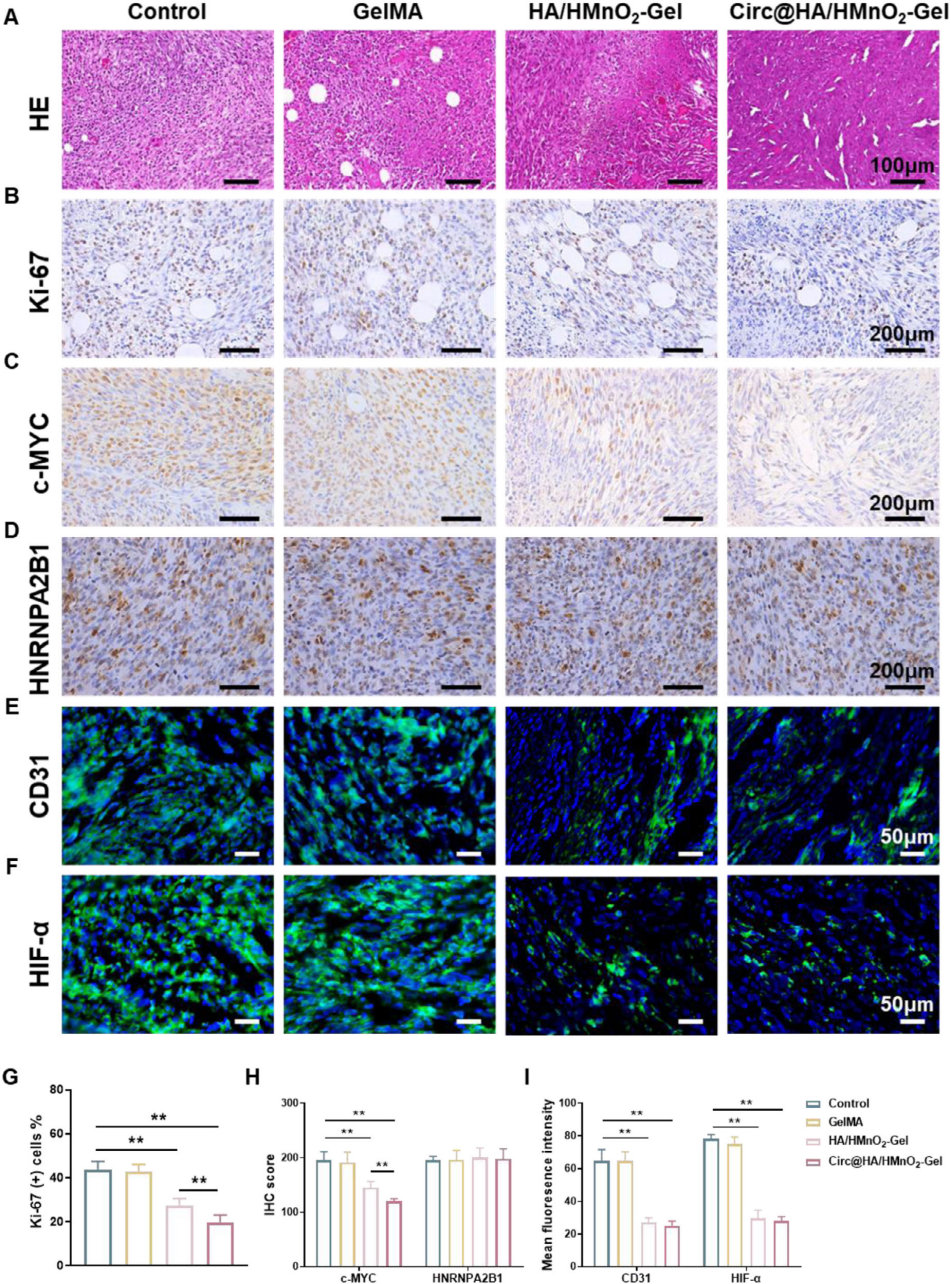
Histological evaluation of the circPSMB1@HA/HMnO_2_‐GelMA composite hydrogel system demonstrates therapeutic efficacy in chordoma models. (A) Representative H&E staining images of chordoma with different treatment. (B) Representative IHC staining images of Ki‐67. (C) Representative IHC staining images of c‐MYC. (D) Representative IHC staining images of HNRNPA2B1. (E) Representative IF staining images of CD31. (F) Representative IF staining images of HIF‐α. (G) Quantitative analysis of IHC staining for Ki‐67 in (B). (H) Quantitative analysis of IHC staining for c‐MYC and HNRNPA2B1 in (C and D). (I) Quantitative analysis of mean fluorescence intensity of CD31 and HIF‐α in (E and F). N = 3 biologically independent samples, *p < 0.05; **p < 0.01.

Histological analyses were further performed to evaluate the antitumor effects of circ@HA/HMnO_2_‐Gel at the tissue level. H&E staining of tumor sections revealed the presence of necrotic regions in both the HA/HMnO_2_‐Gel and circ@HA/HMnO_2_‐Gel groups (Figure 8A). As expected, immunohistochemical analyses showed that the expression levels of Ki67 and c‐MYC in tumors from the circ@HA/HMnO_2_‐Gel group were markedly lower than those in the control, GelMA, and HA/HMnO_2_‐Gel groups (Figure 8B,C), whereas HNRNPA2B1 expression remained unchanged (Figure 8D). In addition, immunofluorescence staining demonstrated that the expression of CD31 and HIF‐α (green fluorescence) was significantly reduced in the HA/HMnO_2_‐Gel and circ@HA/HMnO_2_‐Gel groups (Figure 8E, F), indicating that HMnO_2_ nanoparticles alleviated the hypoxic tumor microenvironment and inhibited tumor angiogenesis. Quantitative analyses of IHC and IF staining further corroborated these observations (Figure 8G–I). In summary, circ@HA/HMnO_2_‐Gel suppresses chordoma growth in vivo by inhibiting tumor cell proliferation and invasion, remodeling the hypoxic tumor microenvironment, and suppressing tumor angiogenesis. In the present study, we systematically characterized the circRNA expression landscape in chordoma and identified circPSMB1 as a tumor‐suppressive circRNA that is significantly downregulated in chordoma tissues and associated with patient prognosis. Our findings establish circPSMB1 as a critical regulator of chordoma progression and uncover a novel m6A‐dependent circRNA‐RBP‐oncogene regulatory mechanism.

## 3 Discussion

Chordoma is a rare but highly aggressive malignant bone tumor characterized by strong local invasiveness, frequent postoperative recurrence, and limited responsiveness to conventional chemotherapy and radiotherapy [23, 24]. Elucidating the molecular mechanisms underlying chordoma progression and identifying effective therapeutic strategies remain critical clinical challenges. In the present study, we systematically characterized a previously unrecognized m6A‐modified circular RNA, circPSMB1, and demonstrated its pivotal tumor‐suppressive role in chordoma. Furthermore, we developed an injectable, tumor‐responsive nanohydrogel system for circPSMB1 delivery, providing both mechanistic insight and translational potential for chordoma therapy.

Our study first identified circPSMB1 as a significantly downregulated circRNA in chordoma tissues compared with adjacent normal tissues. Importantly, reduced circPSMB1 expression was closely associated with shorter continuous disease‐free survival, suggesting its potential value as a prognostic biomarker. CircPSMB1 was confirmed to be generated by back‐splicing of exons 2–5 of the PSMB1 gene and exhibited markedly higher stability than its linear counterpart, consistent with the intrinsic resistance of circular RNAs to exonuclease‐mediated degradation. Functional assays demonstrated that circPSMB1 overexpression significantly inhibited chordoma cell migration, proliferation, and invasion, whereas circPSMB1 knockdown produced the opposite effects. Notably, these phenotypic changes occurred independently of PSMB1 mRNA or protein expression, indicating that circPSMB1 exerts its biological function as a noncoding regulatory RNA rather than through its host gene.

Accumulating evidence indicates that N6‐methyladenosine (m6A) modification plays a crucial role in regulating the fate and function of both linear and circular RNAs [25, 26, 27, 28]. In this study, bioinformatic prediction and experimental validation revealed that circPSMB1 contains a predicted high‐confidence m6A consensus motif, and that its m6A enrichment is dependent on METTL3. Silencing METTL3 or pharmacologically inhibiting methylation significantly reduced m6A‐modified circPSMB1 levels, supporting a direct regulatory relationship. Importantly, METTL3 depletion abolished the interaction between circPSMB1 and the m6A reader protein HNRNPA2B1, supporting an important role for m6A modification in facilitating the association of circPSMB1 with HNRNPA2B1. These findings are consistent with previous reports showing that m6A‐dependent recognition by HNRNPA2B1 serves as a key mechanism by which modified RNAs exert post‐transcriptional regulatory effects [29, 30]. Although these findings support an important role for m6A in facilitating circPSMB1‐HNRNPA2B1 association, the precise m6A site responsible for this interaction remains to be established by site‐directed mutagenesis.

Previous studies have established HNRNPA2B1 as a multifunctional RNA‐binding protein involved in diverse post‐transcriptional processes, including RNA processing, stability, localization, and, in certain contexts, translational regulation [31, 32, 33]. In the present study, circPSMB1 altered c‐MYC protein expression without affecting c‐MYC mRNA abundance and modulated the association between HNRNPA2B1 and c‐MYC mRNA, supporting an HNRNPA2B1‐dependent post‐transcriptional regulatory mechanism. However, the precise downstream mechanism by which HNRNPA2B1 influences c‐MYC protein expression, including whether it directly regulates translational efficiency, remains unclear.

In the present study, we demonstrated that m6A‐modified circPSMB1 competitively binds to HNRNPA2B1, thereby reducing the interaction between HNRNPA2B1 and c‐MYC mRNA. Although circPSMB1 modulation did not significantly alter c‐MYC mRNA abundance, it markedly affected c‐MYC protein expression, indicating that circPSMB1 regulates c‐MYC expression through an HNRNPA2B1‐dependent post‐transcriptional mechanism. Given that c‐MYC is aberrantly activated in more than 70% of human cancers and functions as a master regulator of cell proliferation, metabolism, and survival [34, 35], reduced c‐MYC protein expression provides a plausible explanation for the tumor‐suppressive effects of circPSMB1. Notably, disruption of METTL3‐mediated m6A modification abolished the ability of circPSMB1 to interfere with HNRNPA2B1 binding and attenuate c‐MYC expression, further highlighting the essential role of the METTL3‐m6A‐HNRNPA2B1 regulatory axis in chordoma progression. However, the precise molecular events downstream of HNRNPA2B1, including whether it directly affects ribosome recruitment or translation initiation of c‐MYC mRNA, remain to be further elucidated. Because polysome profiling or ribosome profiling was not performed, the present data do not directly establish altered c‐MYC translational efficiency. Future studies using polysome profiling or ribosome footprinting will provide additional insights into the detailed translational regulation mediated by this axis.

Beyond mechanistic insights, a major strength of this study lies in its translational relevance. Given the high local recurrence rate of chordoma and the limited efficacy of systemic therapies, localized and sustained therapeutic strategies are particularly attractive. We therefore developed an injectable circPSMB1‐loaded nanohydrogel system (circ@HA/HMnO_2_‐Gel) designed for local tumor administration. The hollow mesoporous MnO_2_ nanocarrier enabled high circPSMB1 loading capacity, while hyaluronic acid coating enhanced tumor retention and cellular uptake. Moreover, HMnO_2_ nanoparticles respond to the tumor microenvironment by reacting with glutathione and hydrogen peroxide, generating Mn^2^^+^ and reactive oxygen species, thereby further promoting antitumor effects through oxidative stress [36, 37]. In addition, because the experimental design did not include free circPSMB1 or circPSMB1 delivered through an MnO_2_‐free carrier, the relative contribution of each therapeutic component and the presence of formal therapeutic synergy could not be determined.

Both in vitro and in vivo experiments demonstrated that circ@HA/HMnO_2_‐Gel exhibited favorable biocompatibility and significantly suppressed chordoma growth. In xenograft models, circ@HA/HMnO_2_‐Gel markedly reduced tumor volume and weight compared with control treatments, accompanied by decreased Ki‐67 and c‐MYC expression. In addition, reduced CD31 and HIF‐1α signals indicated impaired angiogenesis and attenuation of hypoxia‐associated signaling, suggesting that the nanohydrogel not only inhibits tumor cell proliferation but also remodels the tumor microenvironment. These findings highlight the therapeutic advantage of combining circRNA‐based gene regulation with tumor‐responsive nanomaterials.

Despite these promising results, several limitations should be acknowledged. First, although MeRIP‐qPCR demonstrated that circPSMB1 undergoes METTL3‐dependent m6A modification, the precise m6A site responsible for HNRNPA2B1 recognition was not experimentally validated by site‐directed mutagenesis. In addition, the absence of detectable circPSMB1 enrichment in the METTL14 RIP fraction does not exclude transient or indirect participation of METTL14 in the methyltransferase complex. Second, the precise translational consequences of HNRNPA2B1 binding remain unresolved because polysome or ribosome profiling was not performed. Third, quantitative biodistribution and pharmacokinetic analyses were not performed. Although the hydrogel was locally administered, systemic redistribution and clearance of the nanomaterial remain to be determined. Fourth, component‐specific controls were insufficient to establish formal therapeutic synergy. Finally, the long‐term efficacy and safety of the platform require validation in orthotopic or postoperative recurrence models.

In conclusion, this study identifies circPSMB1 as an m6A‐modified, tumor‐suppressive circRNA that inhibits chordoma progression through competitive binding of HNRNPA2B1 and subsequent reduction in c‐MYC protein expression through an HNRNPA2B1‐dependent post‐transcriptional mechanism. Moreover, we demonstrate that local delivery of circPSMB1 via a tumor‐responsive nanohydrogel represents a promising therapeutic strategy for chordoma. These findings not only expand the understanding of circRNA‐mediated epigenetic regulation in chordoma but also provide a rational framework for the development of circRNA‐based nanotherapeutics for locally aggressive tumors.

## 4 Materials and Methods

### 4.1 Patients and Tissue Specimen Collection

Tissue samples from 20 patients with chordoma who underwent surgical procedures were analyzed in this study. All participants provided written informed consent, and the study was conducted in accordance with the principles outlined in the Declaration of Helsinki. The study protocol was approved by the Institutional Ethics Committee of The First Affiliated Hospital of Soochow University.

### 4.2 RNA Sequencing

Total RNA was extracted from freshly frozen chordoma tissues and paired adjacent normal tissues using TRIzol reagent (Invitrogen, USA) following the manufacturer's protocol. A cDNA library was generated using an mRNA‐seq sample preparation kit, and high‐throughput sequencing was subsequently performed on an Illumina platform (Illumina) at General Biotechnology. Differential expression analysis was conducted using standard statistical pipelines, with |log2 fold change| ≥ 1 and adjusted *p* <0.05 considered statistically significant. Functional enrichment analyses, including Gene Ontology and pathway enrichment, were performed using the OECloud tools at https://cloud.oebiotech.com.

### 4.3 Cell Culture

Human chordoma cell lines MUG‐Chor1 and U‐CH1 were purchased from the American Type Culture Collection (ATCC). Cells were maintained in a mixed medium of IMDM and RPMI (4:1, v/v) supplemented with 10% fetal bovine serum and 1% penicillin‐streptomycin. Cells were incubated at 37°C in a humidified atmosphere with 5% CO_2_. Cells were routinely passaged using trypsin‐EDTA upon reaching 70%–80% confluence and were periodically tested to exclude mycoplasma contamination.

### 4.4 RNase R Treatment

To confirm the circular structure of circPSMB1 in two chordoma cell lines, total RNA (2 µg) was incubated with RNase R (3 U/µg RNA) at 37°C for 30 min to selectively degrade linear RNAs. Untreated RNA served as a negative control. Following digestion, RNA was purified and subjected to RT‐qPCR analysis to compare the resistance of circPSMB1 and linear PSMB1 mRNA to RNase R treatment. The experiment was independently repeated three times.

### 4.5 Actinomycin D Assay

To assess RNA stability, chordoma cells (U‐CH1 and MUG‐Chor1) were treated with actinomycin D (5 µg/mL) to inhibit de novo transcription. Cells were harvested at indicated time points (0, 4, 8, 12, and 24 h) after treatment. Total RNA was extracted and analyzed by RT‐qPCR to determine the decay rates of circPSMB1 and linear PSMB1 transcripts. Three independent experiments were performed.

### 4.6 Cell Counting Kit‐8 (CCK‐8) Proliferation Assay

Cell proliferation was assessed using the Cell Counting Kit‐8 (CCK‐8, Dojindo Molecular Technology, Japan). Chordoma cells (U‐CH1 and MUG‐Chor1) were seeded into 96‐well plates at a density of 1 × 10^3^ cells per well and transfected as indicated. At designated time points, CCK‐8 reagent was added to each well and incubated for 2 h at 37°C. Absorbance at 450 nm was measured using a microplate reader to assess cell viability. Three independent biological replicates were performed, with three technical replicates for each condition.

### 4.7 5‐Ethynyl‐2′‐Deoxyuridine (EdU) Assay

Cell proliferation was evaluated using an EdU incorporation assay kit BeyoClick EdU Cell Proliferation Kit with Alexa Fluor 488 (Beyotime, Shanghai, China). Briefly, cells (U‐CH1 and MUG‐Chor1) were incubated with EdU working solution for 6 h with different transfected treatments. Cells were then fixed with 4% paraformaldehyde for 30 min, permeabilized in 0.3% Triton X‐100 for 10 min, and incubated with the Click reaction cocktail for 30 min. Subsequently, nuclei were counterstained with Hoechst 33342 for 10 min, and representative fluorescence images were captured using a fluorescence microscope. The cell proliferation rate was calculated using the ratio of EdU‐positive cells (green) to Hoechst‐positive cells (blue). The experiment was independently repeated three times and at least three random fields were analyzed.

### 4.8 Colony Formation Assay

Transfected cells (U‐CH1 and MUG‐Chor1) were seeded into 6‐well plates at low density (500‐1000 cells per well) and cultured for 14 days. Colonies were fixed with paraformaldehyde, stained with crystal violet, and counted. Colonies containing more than 50 cells were considered valid. The assay was independently performed three times.

### 4.9 Wound Healing Assay

Cell migratory ability was evaluated using a scratch wound‐healing assay. Briefly, U‐CH1 and MUG‐Chor1 cells subjected to the indicated transfection conditions were seeded into 6‐well plates at a density of 5 × 10^5^ cells/mL. After reaching appropriate confluence, a straight wound was created with a sterile pipette tip, and floating cells were gently removed by washing with PBS. Wound areas were photographed using an inverted microscope at 0 h and 48 h. For quantification, the remaining wound width (gap area) was measured with ImageJ software, and migration was expressed as the percentage of wound closure. Three independent experiments were performed.

### 4.10 Transwell Migration Assay

The migration capacity of U‐CH1 and MUG‐Chor1 cells with different transfected treatments were also tested by Transwell chamber (8‐µm pore size membrane, 24‐well culture plate). Cells were resuspended in serum‐free medium to a concentration of 1 × 10^5^ cells/mL, and 100 µL of the cell suspension was added to the upper chamber. In contrast, 500 µL of complete medium was added to the lower chamber. After 48 h, cells remaining in the upper chamber were removed using a cotton swab. Cells that traversed the membrane were fixed in 4% paraformaldehyde for 30 min, followed by crystal violet staining (Beyotime, Shanghai, China) for 20 min. Images were captured from five randomly chosen fields under a microscope, and the number of migrated cells was quantified using ImageJ software. Three independent biological replicates were performed.

### 4.11 RT‐qPCR

Total RNA was isolated with FreeZol Reagent (R711, Vazyme, Nanjing, China) following the manufacturer's protocol. RNA purity and quantity were assessed by recording absorbance values at 260 and 280 nm using a NanoDrop spectrophotometer (NanoDrop Technologies, USA). cDNA synthesis was carried out using HiScript III All‐in‐one RT SuperMix (R333, Vazyme, Nanjing, China). Quantitative real‐time PCR was performed on a Bio‐Rad Real‐time PCR Detection System (Bio‐Rad, USA) with SYBR Green Master Mix (Vazyme, Nanjing, China). Relative transcript levels were calculated using the 2^−ΔΔCt^ method, and primer information is listed in Table S1. All RT‐qPCR analyses were performed using three independent biological replicates.

### 4.12 Nuclear and Cytoplasmic Fractionation

Nuclear and cytoplasmic RNA fractions were prepared using a commercial separation kit following the manufacturer's instructions (Cytosolic and Nuclear RNA Extraction System, 21000, Norgen Biotek, Canada). The relative distribution of RNA in each compartment was subsequently quantified by RT‐qPCR, with U3 and GAPDH serving as nuclear and cytoplasmic reference markers, respectively. The experiment was independently repeated three times.

### 4.13 RNA Fluorescence in Situ Hybridization (RNA‐FISH)

RNA‐FISH was performed with a circPSMB1‐specific probe to visualize its intracellular localization. The oligonucleotide‐modified probe was synthesized by GenePharma (Shanghai, China), and hybridization/staining procedures were carried out using an RNA Fluorescence In Situ Hybridization Kit (Beyotime, China). After probe hybridization, nuclei were counterstained with DAPI, and fluorescence signals were captured using a confocal microscope. Representative images from three independent experiments are shown. The circPSMB1 probe sequence was as follows: 5′‐FAM‐CAGCAATTGCCAGTATAGTACTGGTTGTCAAGCAGGGGCT‐3′.

### 4.14 RNA Interference and Transfection

The circPSMB1 overexpression construct, shRNAs targeting circPSMB1, and METTL3‐specific siRNAs were obtained from GenePharma (Shanghai, China). Transfections were conducted using Lipomaster 3000 reagent (TL301, Vazyme, Nanjing, China) according to the manufacturer's guidelines. For stable gene modulation, lentiviral particles carrying circPSMB1 overexpression plasmids or shRNA vectors were generated and used for cell transduction. Target sequences are listed in Table S2.

### 4.15 Western Blot

Cells and tissue samples were collected and lysed with RIPA buffer containing proteinase inhibitor. The protein concentration was measured with BCA Protein Assay Kit (Beyotime, P0012, China). The protein samples were separated on SDS‐PAGE and subsequently transferred onto nitrocellulose membranes (0.45 µm, Millipore, USA). After blockage, the membranes were incubated at 4°C overnight with primary antibodies including c‐MYC (1:1000 dilution, Cat No. 10828‐1‐AP, ProteinTech), HNRNPA2B1 (1:2000 dilution, Cat No. 14813‐1‐AP, ProteinTech), GAPDH (1:10000 dilution, Cat No. 60004‐1‐Ig, ProteinTech). After thorough TBST washing, peroxidase‐conjugated (HRP)‐linked secondary antibody (Goat Anti‐Rabbit IgG, 1:1000 dilution, Cat No. A0208, Goat Anti‐Mouse IgG, 1:1000 dilution, Cat No. A0216, Beyotime, China) was used to incubate with these membranes for 1 h at room temperature. Subsequently, excess secondary antibodies were eliminated through multiple TBST washes. The antigen‐antibody reaction was visualized via an ECL kit (Thermo Fisher Scientific, USA) and imaged by a chemiluminescence detection system. Quantitative analysis of the immunoblots was performed using ImageJ software. Western blot analyses were independently repeated three times. Representative blots are shown.

### 4.16 Silver Staining and Mass Spectrometry

Silver staining was performed using a Rapid Silver Stain Kit (Beyotime, China). Protein identification and quantitative proteomic analysis were conducted by Genelily Biotech Co., LTD. Proteome Discoverer software (v1.4; Thermo Fisher Scientific) was applied for peptide/protein assignment and quantification. Proteins were considered differentially enriched when the circPSMB1‐to‐control ratio was >2 and at least two unique peptides were detected.

### 4.17 RNA Pull‐Down

Biotin‐labeled probes targeting circPSMB1 and the corresponding antisense control were synthesized by GenePharma (Shanghai, China). RNA pull‐down experiments were carried out using the Pierce Magnetic RNA‐Protein Pull‐Down Kit (20164, Thermo Fisher Scientific) in accordance with the manufacturer's protocol. Briefly, approximately 1 × 10^7^ cells were harvested, lysed, and incubated with 100 µL streptavidin‐coated magnetic beads coupled to the biotinylated circPSMB1 probe at 4°C for 30 min with gentle rotation (10 rpm). RNA‐protein complexes captured by the beads were washed, and bound proteins were eluted and resolved by SDS‐PAGE, followed by silver staining (Fast Silver Stain Kit, Beyotime, China). Differential bands were excised, digested with trypsin at 37°C overnight, and the resulting peptides were dried and reconstituted for LC‐MS/MS analysis (MaxQuant v1.6.17.0). Probe sequences were as follows: circPSMB1, 5′‐Biotin‐CTGCTTGACAACCAGTACTATACTGGCAAT‐3′; negative control, 5′‐Biotin‐GTACCTAGTCTGACATCGATGACCTAGTCA‐3′. RNA pull‐down assays were independently repeated three times.

### 4.18 RNA Immunoprecipitation (RIP)

RIP assays were conducted using the Magna RIP Kit (17‐701, Millipore) following the supplier's instructions. U‐CH1 and MUG‐Chor1 cells (1 × 10^7^) were lysed in 1 mL RIP lysis buffer supplemented with RNase inhibitors. The lysates were then incubated overnight at 4°C with magnetic beads pre‐coated with either control IgG or an anti‐HNRNPA2B1 antibody under continuous rotation. Co‐immunoprecipitated RNAs were purified and subsequently quantified by RT‐qPCR. RIP assays were performed in three independent biological replicates.

### 4.19 m6A RNA Immunoprecipitation Followed by RT‐qPCR (MeRIP‐qPCR)

MeRIP‐qPCR was performed using a commercial MeRIP m6A kit (BersinBio, Guangzhou, China) according to the manufacturer's instructions. Briefly, total RNA isolated from U‐CH1 and MUG‐Chor1 cells was fragmented to approximately 300 nt and RNA was incubated with 5 µg of an anti‐m6A antibody validated for m6A RNA immunoprecipitation (MeRIP) applications (Cat No. 68055‐1‐Ig, Proteintech) or control IgG for 2 h at 4°C. The antibody‐RNA complexes were captured using protein A/G magnetic beads, washed, and eluted following proteinase K digestion. The immunoprecipitated RNA was then purified and subjected to RT‐qPCR using circPSMB1‐specific primers. Relative m6A enrichment was calculated by normalizing the MeRIP signal to the corresponding input RNA. All experiments were independently performed three times.

### 4.20 Synthesis of circPSMB1@HA/HMnO_2_‐GelMA

Solid silica nanoparticles (sSiO_2_) were prepared according to a previously published method [38]. Briefly, an aqueous KMnO_4_ solution (300 mg) was slowly introduced dropwise into a suspension containing sSiO_2_ (40 mg) under ultrasonic agitation. After reaction for 6 h, the resulting product was collected by centrifugation at 14,800 rpm. The mesoporous MnO_2_‐coated sSiO_2_ particles were subsequently treated with 2 m Na_2_CO_3_ solution at 60°C for 12 h to remove the silica template. Finally, hollow mesoporous MnO_2_ nanoshells (HMnO_2_) were harvested by centrifugation and thoroughly rinsed with deionized water multiple times. After mixing the HMnO_2_ solution (0.2 mg mL^−^^1^) with circPSMB1‐oe for 12 h, circPSMB1@HMnO_2_ was successfully obtained, loaded with circPSMB1. Subsequently, circPSMB1@HMnO_2_ was added to HA to yield circPSMB1@HA/HMnO_2_. Briefly, 100 mg HA (40 kDa) was dissolved in distilled water (20 mL). Then, the HA was activated under stirring for 30 min in the presence of 1‐ethyl‐3‐(3‐(dimethylamino)propyl)‐carbodiimide (EDC) and N‐hydroxysuccinimide (NHS). Then, the HMnO_2_ NPs were added dropwise at various HA/HMnO_2_ NP weight ratios (w/w) and reacted with activated carboxylic ester of HA under stirring at 35°C for 3 h. Finally, this mixture was added to a 5% GelMA solution to yield the final product circPSMB1@HA/HMnO_2_‐GelMA which was used for further experiments.

### 4.21 Animal Models

For xenograft establishment, U‐CH1 cells (1 × 10^7^ cells per mouse) were injected subcutaneously into the unilateral axillary region of 8‐week‐old male BALB/c nude mice. Two weeks after tumor implantation, mice received intratumoral administration of normal saline, GelMA, HA/HMnO_2_‐Gel, or circ@HA/HMnO_2_‐Gel, and tumor progression under each treatment regimen was monitored. Tumor dimensions were recorded at 5‐day intervals, and volumes were calculated using the formula: volume = 0.5 × length × width [2]. At 30 days after inoculation, animals were sacrificed, and subcutaneous tumors were excised for further evaluation. Five mice were included in each experimental group for the in vivo experiments. All animal procedures were approved by the Institutional Ethics Committee of Soochow University (Approval No. SUDA20251019A05).

### 4.22 Histopathological and Immunohistochemical Analysis

Excised tumor specimens were fixed for 24–48 h and then embedded in either paraffin or optimal cutting temperature (OCT) compound (Sakura Finetek, Torrance, CA). Paraffin sections (5 µm) were prepared for hematoxylin and eosin (H&E) staining as well as immunohistochemistry (IHC). For immunofluorescence (IF) analysis, frozen coronal sections were cut at 10 µm thickness. Primary antibodies against Ki‐67 (1:200, Cat. ab16667, Abcam), HNRNPA2B1 (1:400, Cat. 14813‐1‐AP, ProteinTech), CD31 (1:200, Cat. 11265‐1‐AP, ProteinTech), and HIF‐α (1:400, Cat. ab179483, Abcam) were used for IHC and IF staining. Images were acquired with a Zeiss Axiovert 200 microscope (Carl Zeiss Inc., Thornwood, NY), and signal quantification was performed using ImageJ software. Tumor sections from all animals in each group were analyzed, and at least three random microscopic fields per section were quantified using ImageJ.

### 4.23 Statistical Analysis

Statistical analyses and graph generation in this study were performed using GraphPad Prism software (GraphPad Software Inc., USA). Unless otherwise indicated, all in vitro experiments were independently performed at least three times. Quantitative data are presented as the mean ± SD from three independent biological replicates. Representative images from independent experiments are shown. Comparisons between two groups were performed using Student's t‐test, while comparisons among multiple groups were performed using one‐way analysis of variance (one‐way ANOVA). A *p* value < 0.05 was considered statistically significant.

## Author Contributions

**Yingchuang Tang**: writing – original draft, project administration, funding acquisition, visualization, data curation. **Liang Qiu**: investigation, visualization, validation, data curation, software. **Xingbang Ruan**: validation, investigation, software. **Yihan Shi**: investigation, data curation, software. **Ye Wang**: data curation, validation, investigation. **Hanwen Li**: conceptualization, funding acquisition, writing – review and editing, visualization, validation. **Kai Zhang**: methodology, formal analysis, writing – review and editing, project administration, resources. **Kangwu Chen**: conceptualization, resources, supervision, writing – review and editing.

## Conflicts of Interest

The authors declare no conflicts of interest.

## Ethics Approval

The study was approved by the Ethics Committee of Soochow University, Ethics Approval Number: No. SUDA20251019A05.

## Supporting information




**Supporting File**: advs77417‐sup‐0001‐SuppMat.docx.

## Data Availability

The data that support the findings of this study are available from the corresponding author upon reasonable request.
