# Supplementary material for: Restoration of m6A‐Modified circPSMB1 Suppresses Chordoma Progression and Enables Local Nanotherapeutic Intervention
Source: Adv Sci (Weinh). 2026 Aug 29:e77417. Online ahead of print. doi: 10.1002/advs.77417 (PMC13525617; doi:10.1002/advs.77417)
Supplement: Supplementary file 1 [file ADVS-9999-e77417-s001.docx]

**Supplementary Figures**

**Supplementary Figure 1. Screening of differently expressed circRNAs that inhibit chordoma progression.** Analysis by high-throughput RNA sequencing revealed that, compared with adjacent non-tumor tissues, chordoma tissues exhibited upregulation of 1,461 circRNAs and downregulation of 1,527 circRNAs.

**Supplementary Figure 2. PSMB1 mRNA expression in U-CH1 and MUG-Chor1 cells is unaffected by circPSMB1 expression. (A)** Knockdown of circPSMB1 by shRNA technology in U-CH1 and MUG-Chor1 cells does not affect the expression level of PSMB1 mRNA. **(B)** Exogenous enforced overexpression of circPSMB1 in U-CH1 and MUG-Chor1 cells fails to alter the expression level of PSMB1 mRNA. Data are presented as the mean ± SD from three independent biological replicates.

**Supplementary Figure 3. Prediction of Potential m6A Sites in circPSMB1**. One high-confidence binding pocket, two medium-confidence binding pockets, and two low-confidence binding pockets were identified.

**Supplementary Figure 4. MeRIP-qPCR confirms METTL3-dependent m6A modification of circPSMB1.** Gene-specific MeRIP-qPCR analysis of circPSMB1 in U-CH1 and MUG-Chor1 cells following control or METTL3 knockdown. Data are presented as the mean ± SD from three independent biological replicates. *p < 0.05; **p < 0.01.

**Supplementary Figure 5. METTL3-dependent m6A modification facilitates the interaction between circPSMB1 and HNRNPA2B1.** Treatment with 3-deazaadenosine (DAA) or knockdown of METTL3 significantly reduced the interaction between circPSMB1 and HNRNPA2B1, supporting the requirement of m6A modification for HNRNPA2B1 recognition. Data are presented as the mean ± SD from three independent biological replicates. *p < 0.05; **p < 0.01.

**Supplementary Figure 6.** The expression level of c-MYC mRNA following the knockdown and overexpression of circPSMB1 was examined by RT-qPCR. Data are presented as the mean ± SD from three independent biological replicates.

**Supplementary Figure 7.** Western blot was performed to validate the expression of HNRNPA2B1 protein following the knockdown and overexpression of circPSMB1. Data are presented as the mean ± SD from three independent biological replicates.

**Supplementary Figure 8. Functional rescue experiments demonstrate that c-MYC as a downstream functional effector of circPSMB1. (A)** Representative EdU staining images of U-CH1 and MUG-Chor1 cells transfected with the indicated circPSMB1 and c-MYC overexpression constructs. **(B)** Quantification of EdU-positive cells shown in (A). **(C)** CCK-8 assays evaluating cell proliferation following circPSMB1 overexpression with or without c-MYC restoration. **(D)** Representative images of Transwell migration rescue assays in U-CH1 and MUG-Chor1 cells. **(E)** Quantification of migrated cells shown in (D). Data are presented as the mean ± SD from three independent biological replicates. *p < 0.05; **p < 0.01.

**Supplementary Figure 9. Rheological characterization of circPSMB1@HA/HMnO₂-GelMA hydrogel.** Frequency sweep measurements showing the storage modulus (G′) and loss modulus (G″) of GelMA and circPSMB1@HA/HMnO₂-GelMA hydrogels over a frequency range of 0.1-10 Hz. The incorporation of HA/HMnO₂ nanoparticles and circPSMB1 did not substantially alter the viscoelastic properties of the GelMA hydrogel.

**Supplementary Figure 10. Swelling and degradation properties of circPSMB1@HA/HMnO₂-GelMA hydrogel. (A)** Swelling behavior of GelMA and circPSMB1@HA/HMnO₂-GelMA hydrogels over 12 h. **(B)** In vitro degradation profiles of GelMA and circPSMB1@HA/HMnO₂-GelMA hydrogels over 14 days. The incorporation of HA/HMnO₂ nanoparticles and circPSMB1 exerted minimal effects on the swelling and degradation characteristics of the hydrogel.

**Supplementary Figure 11. Tumor microenvironment-responsive release behavior of circPSMB1@HA/HMnO₂-GelMA hydrogel.** **(A)** Cumulative Mn²⁺ release from circPSMB1@HA/HMnO₂-GelMA hydrogel under physiological conditions (pH 7.4), acidic conditions (pH 5.5), and acidic conditions supplemented with 10 mM GSH (pH 5.5+10 mM GSH). **(B)** Cumulative circPSMB1 release from circPSMB1@HA/HMnO₂-GelMA hydrogel under the same conditions. Acidic pH and elevated GSH levels markedly accelerated the release of both Mn²⁺ and circPSMB1, demonstrating the tumor microenvironment-responsive release behavior of the hydrogel.

**Supplementary Figure 12. Selection of HMnO_2_ concentration. (A**) CCK8 assay for co-culture of U-CH1 cells with different concentrations of HMnO_2_. (**B)** Live/dead staining assay of U-CH1 and MUG-Chor1 cells co-cultured with different treatments (80 µg/mL HMnO_2_). N=3 biologically independent samples, **p* < 0.05; ***p* < 0.01.

**Supplementary Figure 13.** Quantitative analysis of the Cellular uptake assay in figure 5G. N=3 biologically independent samples, **p* < 0.05; ***p* < 0.01.

**Table S1. Sequences of the primers used in this work.**

| Gene symbol | Forward primer | Reverse primer |
| --- | --- | --- |
| hsa-PSMB1_0002 | CCCCTGCTTGACAACCAGTAC | GGGCTATCCCGCGTATGAAT |
| hsa-ACTN2_0004 | CCTAAAATGTTGGATGCTGAAGAAAC | GATGAGGGCACAGAGTCCAA |
| hsa-CACNA2D1_0013 | CCAGAGTTTATCCCAAAGAATTCGGA | TCATTGCAGTAATCTCTTGGTGC |
| hsa-RRAS2_0001 | CAGATCTGGATCATCAAAGACAGTCC | CAAAATATCTAGCCGGGCTGC |
| hsa-CACNA2D1_0078 | GGGAAACTTCTTAAACCTGCAGATTC | TTGCAGTAATCTCTTGGTGCT |
| hsa-MAPT_0007 | GGCGGCAGTGGGG | TTTTGGAGGTTCACCAGAGC |
| hsa-EGF_0001 | ACTTGGGAGCCTGGTCC | CACTGAGACACCAGCATCCA |
| hsa-PPP3CB_0002 | ACTGAATATTTTACCTTTAAGCAGGAATCTG | ACCCTGGGTATCCCATCCAA |
| hsa-NRG4_0009 | GCAGAAAATGAAACACCACTAGGAAG | CCCAGGCTTCAGGTCTCAAC |
| hsa-GFRA1_0001 | CACCGTGTGCAAATTTACAGATCT | GCTGCTGACAGACCTTGACT |
| CircPSMB1-Divergent | CCCCTGCTTGACAACCAGTAC | TTGCTTCAATAATCTTTGTCAGCGT |
| CircPSMB1-Convergent | AGTCATTGGATGCAGCGGTT | CCTCCAGCCTTGAAGGAGTC |
| PSMB1 | TCAGCAAGTGCCATGCTACA | GCGGAACATGCTCCACATTC |
| GAPDH | AGAAGGCTGGGGCTCATTTG | GCAGGAGGCATTGCTGATGAT |
| U3 | AGAAGCCACAAGGAGCAGGC | TGGCGAATGGTACTTGCAGGG |
| β-actin | GAGCTACGAGCTGCCTGACG | TGCCAGGGCAGTGATCTCCT |

**Table S2. Target sequences of the siRNAs, shRNAs and plasmids used in this study.**

| Gene symbol | Targeted sequence |
| --- | --- |
| siMETTL3#1 | 5’- CUUAACAUUGCCCACUGAU tt |
| siMETTL3#2 | 5’-AAGUAUGUUCACUAUGAAA tt |
| siMETTL3#3 | 5’-CAAGGAACAAUCCAUUGUU tt |
| sh-circPSMB1#1 | CCGGAAACCTGAAGTAGTCCGGTCACTCGAGTGACCGGACTACTTCAGGTTTTTTTTT |
| sh-circPSMB1#2 | CCGGAGAAACCTGAAGTAGTCCGGTCTCGAGACCGGACTACTTCAGGTTTCTTTTTTT |
| sh-circPSMB1#3 | CCGGACCTGAAGTAGTCCGGTCAAGCTCGAGCTTGACCGGACTACTTCAGGTTTTTTT |
| oe-circPSMB1 | AGTAGAGACGGGGTTTCACCATGTTGGCCAGGCTGGTCTTCACTTTTTGTAAAGGTACGTACTAATGACTTTTTTTTTATACTTCAGTACTATACTGGCAATTGCTGGAGAAGATTTTGCAATTGTTGCTTCTGATACTCGATTGAGTGAAGGGTTTTCAATTCATACGCGGGATAGCCCCAAATGTTACAAATTAACAGACAAAACAGTCATTGGATGCAGCGGTTTTCATGGAGACTGTCTTACGCTGACAAAGATTATTGAAGCAAGACTAAAGATGTATAAGCATTCCAATAATAAGGCCATGACTACGGGGGCAATTGCTGCAATGCTGTCTACAATCCTGTATTCAAGGCGCTTCTTTCCATACTATGTTTACAACATCATCGGTGGACTTGATGAAGAAGGAAAGGGGGCTGTATACAGCTTTGATCCAGTAGGGTCTTACCAGAGAGACTCCTTCAAGGCTGGAGGCTCAGCAAGTGCCATGCTACAGCCCCTGCTTGACAACCAGGTAAGAAGCAAGGAAAAGAATTAGAGACCAGCCTGGCCAACATGGTGAAACCTTGTCTCTACT |
